# Aspidoptoids A–D: Four New Diterpenoids from *Aspidopterys obcordata* Vine

**DOI:** 10.3390/molecules25030529

**Published:** 2020-01-25

**Authors:** Peng Sun, Dong-Hua Cao, Yi-Dian Xiao, Zong-Yi Zhang, Jia-Nan Wang, Xiao-Cui Shi, Chun-Fen Xiao, Hua-Bin Hu, You-Kai Xu

**Affiliations:** 1 CAS Key Laboratory of Tropical Plant Resources and Sustainable Use, Xishuangbanna Tropical Botanical Garden, Chinese Academy of Sciences, Menglun 666303, China; sunpeng@xtbg.ac.cn (P.S.); caodonghua@xtbg.ac.cn (D.-H.C.); zhangzongyi@xtbg.ac.cn (Z.-Y.Z.); wangjianan@xtbg.ac.cn (J.-N.W.); shixiaocui@xtbg.ac.cn (X.-C.S.); xiaocf@xtbg.ac.cn (C.-F.X.); huhb@xtbg.ac.cn (H.-B.H.); 2 University of Chinese Academy of Sciences, Beijing 100049, China; 3 School of Chemical Science and Technology, Key Laboratory of Medicinal Chemistry for Nature Resources, Ministry of Education, Yunnan University, Kunming 650019, China; xiaoyidian@mail.kib.ac.cn; 4 State Key Laboratory of Phytochemistry and Plant Resources in West China, Kunming Institute of Botany, Chinese Academy of Sciences, Kunming 650201, China

**Keywords:** *Aspidopterys obcordata*, Malpighiaceae, norditerpeniod, cytotoxic activity, anti-inflammatory activity

## Abstract

Four new diterpenoids, named aspidoptoids A–D (**1**–**4**), together with two known analogues (**5**–**6**) were isolated from *Aspidopterys obcordata* vine. Aspidoptoids A–B (**1**–**2**) are the first examples of phenylethylene-bearing 20-nor-diterpenoids of which aspidoptoid B (**2**) possesses a rare 3,10-oxybridge. Their structures and absolute configuration were determined by extensive spectroscopic analyses (IR, HRESIMS, 1D and 2D NMR) and electronic circular dichroism (ECD) calculation. In addition, all the isolates were evaluated for their cytotoxic activities and inhibitory effects on the nitric oxide (NO) production.

## 1. Introduction

**Figure 1:**
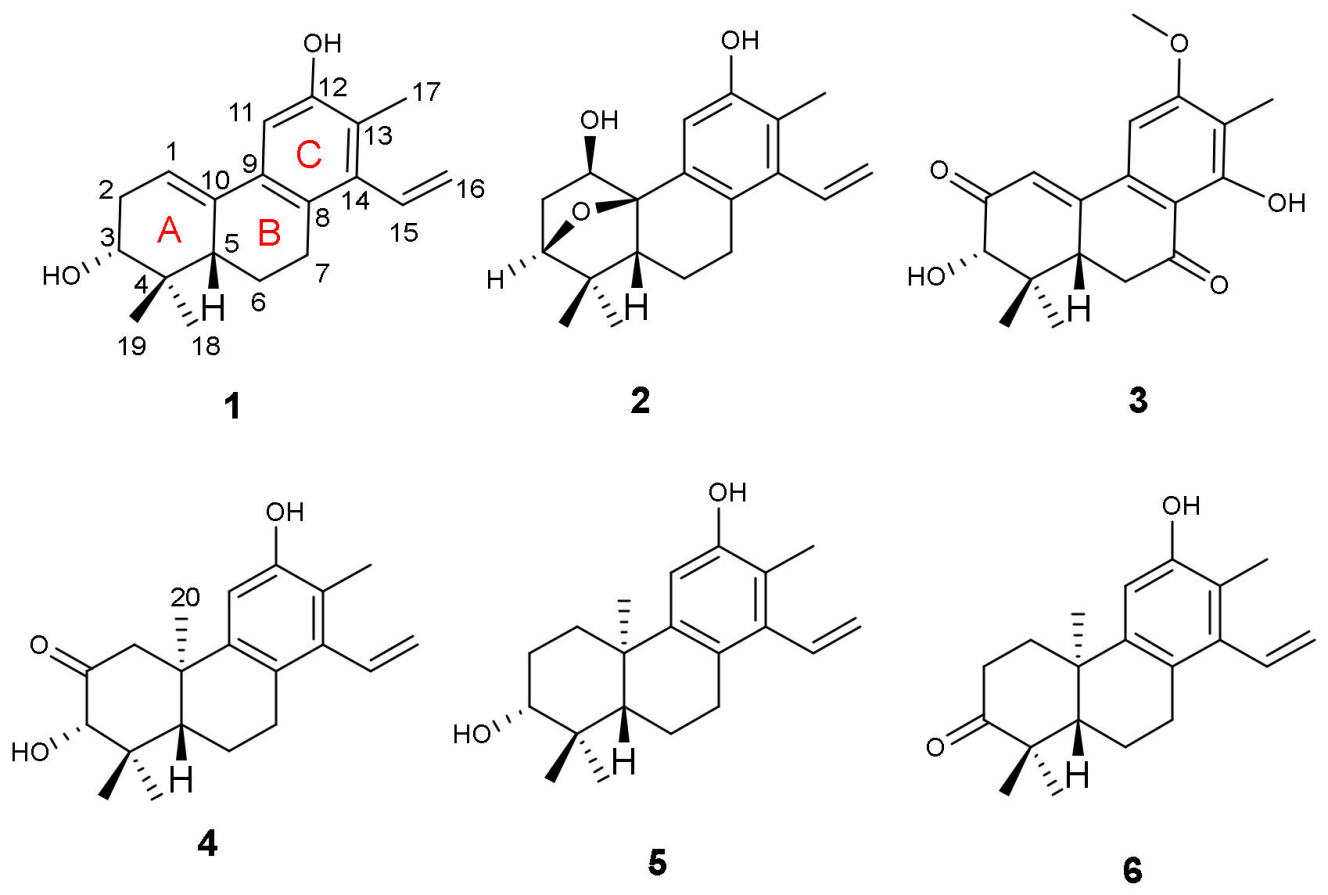
The structures of compounds **1**–**6**.

*Aspidopterys* genus (Malpighiaceae family) has approximately 20 species, mainly distributed in the regions of tropical Asia, of which nine species and one variant grow in China. *Aspidopterys obcordata* Hemsl is a woody liana in 600–1600 m habitats including sparse forests, valley shrub forests, or mountains in Hainan and Yunnan provinces, China [1]. The stems and branches of *A. obcordata* have been traditionally used as folk medicine by Dai people in Xishuangbanna, Southwest China, for treating various diseases such as urinary tract infections, acute and chronic nephritis, cystitis, pyelitis, urinary calculi, rheumatic bone pain, postpartum body deficiency, and anorexia [2,3]. To date, just a few phytochemical and pharmacological studies have been conducted on this plant. Previous phytochemical investigations on *A. obcordata* led to the isolation of triterpenes, sterols, tannins, and polyoxypregnane glycosides [4,5,6,7]. Among them, polyoxypregnane glycosides have been reported to exhibit anti-urolithiatic [8] and anti-tumor activities [6]. In continuing the search for structurally diverse and biologically interesting metabolites from Dai medicine plants, the chemical components of *A. obcordata* vine were investigated. As a result, four new diterpenoids, named aspidoptoids A–D (**1**–**4**), together with two known analogues (**5**–**6**) were isolated from the 95% EtOH extract of *A. obcordata* vine. Aspidoptoids A–B (**1**–**2**) are the first example of phenylethylene-bearing 20-nor-diterpenoid of which aspidoptoids B (**2**) possesses a rare 3,10-oxybridge (Figure 1). All the isolates were tested for their cytotoxic activities against human myeloid leukemia (HL-60), hepatocellular carcinoma (SMMC-7721), lung cancer (A-549), breast cancer (MCF-7), and colon cancer (SW480) cell lines and NO inhibitory effects on lipopolysaccharide (LPS)-stimulated RAW 264.7 macrophages cells. Herein, we report the isolation, structure elucidation, and biological evaluation of these compounds.

## 2. Results and Discussion

**Figure 2:**
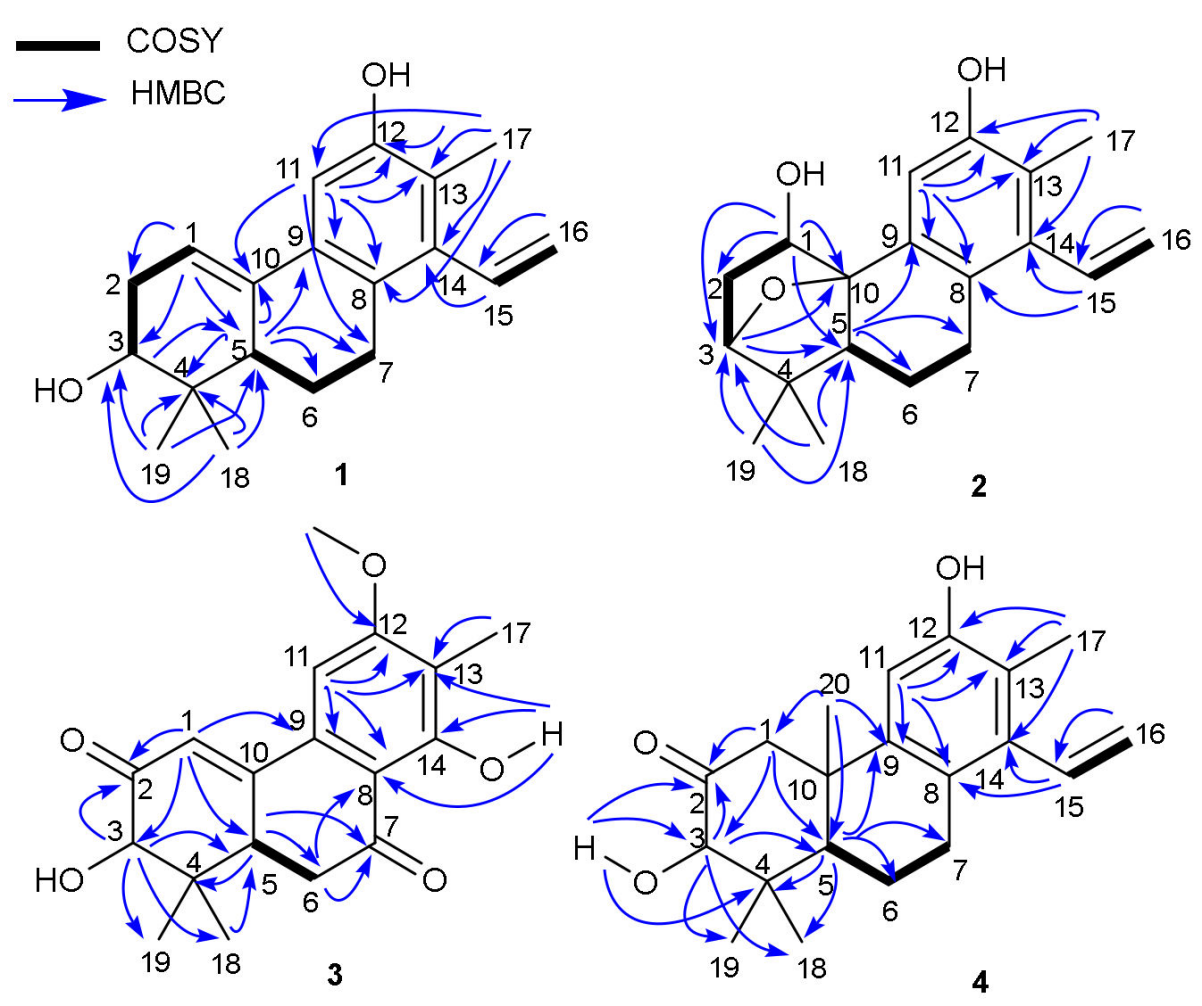
Selected ^1^H-^1^H COSY and HMBC correlations of compounds **1**–**4**.

Compound **1**, a yellow powder, possessed a molecular formula C_19_H_24_O_2_ as determined by the negative HRESIMS peak at *m*/*z* 283.1704 [M − H]^−^ (calcd. for C_19_H_23_O_2_, 283.1704), corresponding to eight degrees of unsaturation. The IR absorptions indicated the presence of hydroxyl (3430 cm^−1^) and olefinic (1630 cm^−1^) groups. The 1D NMR spectra of **1** displayed the occurrence of 19 carbon signals which were further classified by the DEPT experiment as three methyls, four methylenes (one olefinic), five methines (three olefinic and one oxygenated), and seven quaternary carbons (six olefinic and one aliphatic) (Table 1 and Table 2). In addition, a terminal vinyl (*δ*_H_ 6.61, dd, *J* = 17.9, 11.4 Hz, H-15; *δ*_H_ 5.55, dd, *J* = 11.4, 2.0 Hz, H-16*α*; *δ*_H_ 5.17, dd, *J* = 17.9, 2.0 Hz, H-16*β*; *δ*_C_ 135.3 and 120.1), three singlet methyls (*δ*_H_ 2.19, 1.11, and 0.77, all s; *δ*_C_ 24.4, 13.4, and 13.1), a trisubstituted double bond (*δ*_H_ 6.18, t, *J* = 3.1 Hz; *δ*_C_ 134.5 and 116.5) and one penta-substituted benzene ring (*δ*_H_ 7.03, s; *δ*_C_ 152.1, 139.5, 132.9, 127.3, 121.3, and 108.3) were easily distinguished by analysis of the NMR data (Table 1 and Table 2). Those identified functionalities accounted for 5 out of 8 degrees of unsaturation; the remaining required three additional rings in **1**. Comparing its 1D NMR data with those of spruceanol (**5**), a cleistanthane diterpenoid reported from *Cunuria spruceana* [9], indicated that they had the very similar rings A, B, and C, with the differences being the presence of Δ^1,10^ double bond in **1** and the absence of CH_2_-1 (*δ*_H_ 3.38, t, *J* = 6.8 Hz; *δ*_C_ 25.4) and CH_3_-20 (*δ*_H_ 1.19, s; *δ*_C_ 24.8) in **5**. The proposition was further confirmed by 2D NMR experiments. The ^1^H-^1^H COSY correlations constructed two partial structures of –CH–CH_2_–CH(O)– and –CH–CH_2_–CH_2_– in **1** as shown with bold lines in Figure 2. Moreover, the HMBC correlations (Figure 2) of both methyl protons at *δ*_H_ 0.77 (H_3_-18) and 1.11 (H_3_-19) with C-3 (*δ*_C_ 74.9)/C-4 (*δ*_C_ 37.2)/C-5 (*δ*_C_ 46.2); another methyl at *δ*_H_ 2.19 (H_3_-17) with C-8 (*δ*_C_ 127.3)/C-11 (*δ*_C_ 108.3)/C-12 (*δ*_C_ 152.1)/C-13 (*δ*_C_ 121.3)/C-14 (*δ*_C_ 139.5); the aliphatic methine at *δ*_H_ 2.02 (H-5) with C-4 (*δ*_C_ 37.2)/C-6 (*δ*_C_ 24.0)/C-7 (*δ*_C_ 28.9)/C-9 (*δ*_C_ 132.9)/C-10 (*δ*_C_ 134.5); the aromatic proton at *δ*_H_ 7.03 (H-11) with C-7/C-8/C-9/C-10/C-12/C-13 furnished the typical fused A/B/C-ring system of the diterpenoid core like spruceanol. The location of the Δ^1,10^ double bond was further confirmed by HMBC correlations from H-1 (*δ*_H_ 6.18) to C-2 (32.3)/C-3 (*δ*_C_ 74.9)/C-5 (*δ*_C_ 46.2)/C-10 (*δ*_C_ 134.5), combined with ^1^H-^1^H COSY correlations H-1 (*δ*_H_ 6.18, t, *J* = 3.1 Hz) with H-2 (*δ*_H_ 2.16, ddd, *J* = 10.2, 6.5, 3.1 Hz and *δ*_H_ 2.47, m). These observations suggested **1** to be the first example of phenylethylene-bearing 20-nor-diterpenoid with a Δ^1,10^ double bond. 

**Figure 3:**
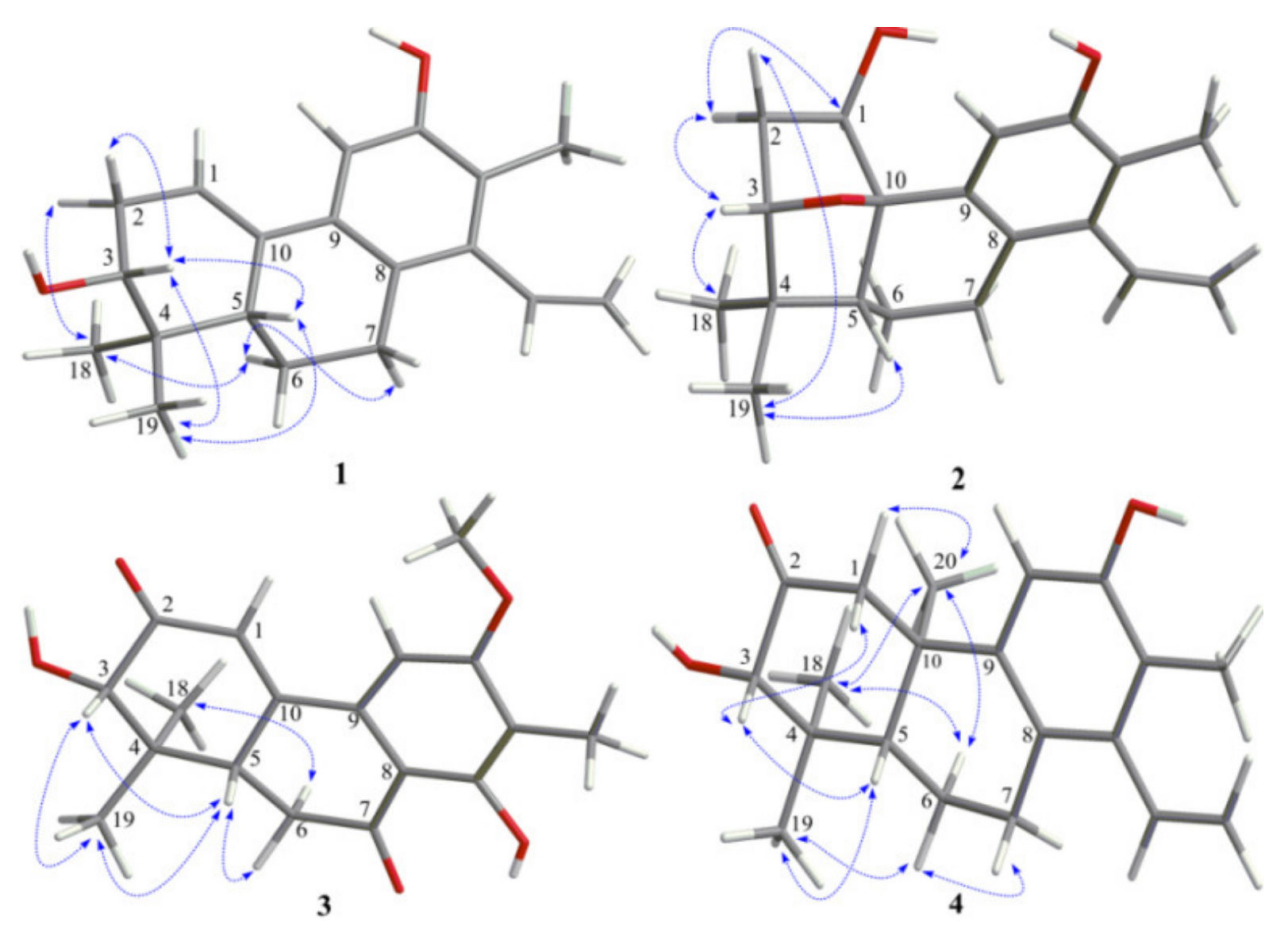
Key ROESY correlations of compounds **1**–**4**.

**Figure 4:**
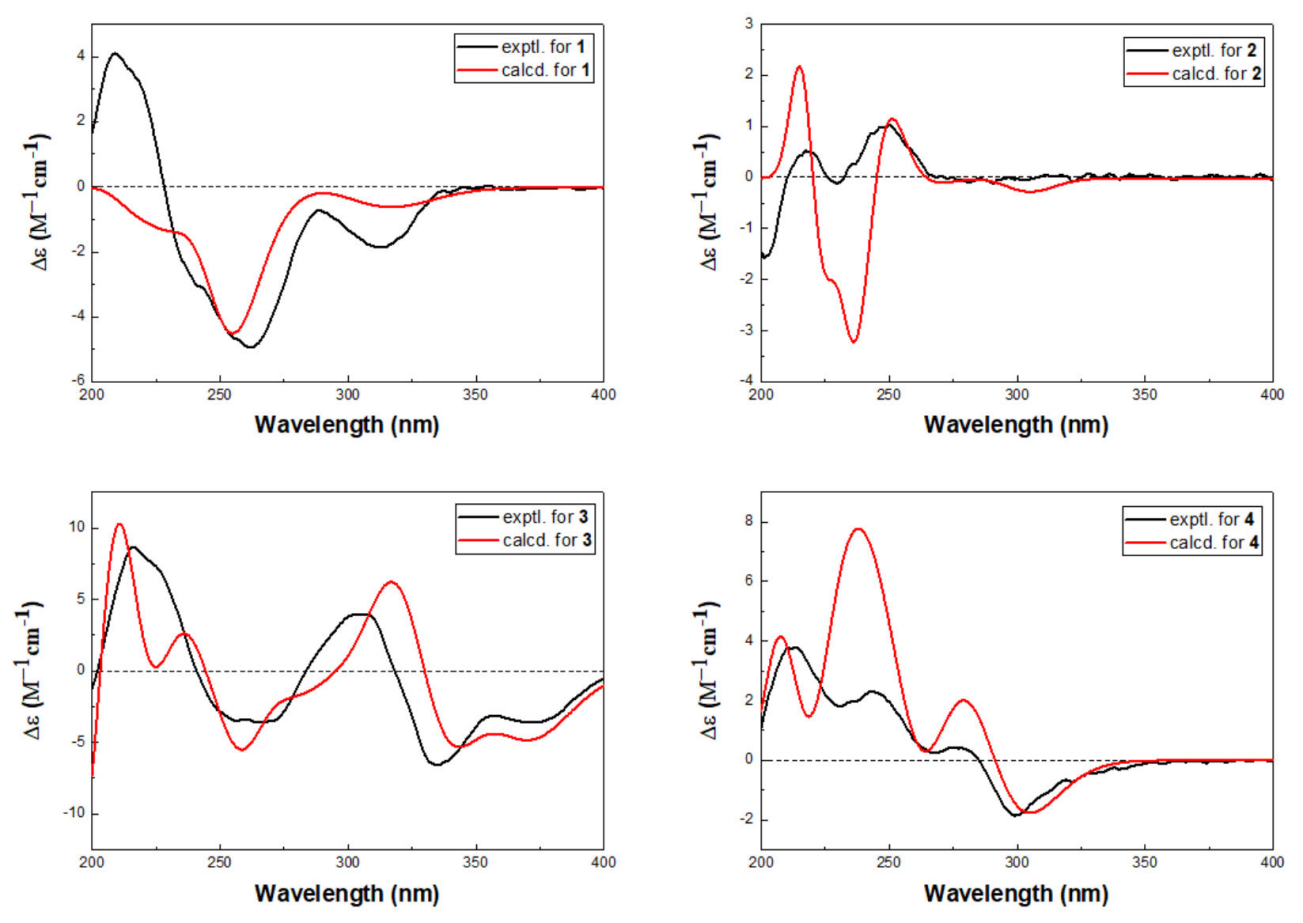
Experimental and calculated ECD spectra of compounds **1**−**4**.

The relative configuration of **1** was proposed on the basis of ROESY data (Figure 3) and by comparison with that of spruceanol. The ROESY correlations of H-2*α*/H_3_-18, H-6*α*/H-7*α*, and H-6*α*/H_3_-18 indicated that these protons adopt the same orientation, whereas those of H-2*β/*H-3, H-3/H-5, H-3/H_3_-19, and H-5/H_3_-19 placed these protons on the opposite face of the ring system. The absolute configuration of **1** was determined by quantum chemical TDDFT calculation of its theoretical ECD spectrum. In the 200–400 nm regions, both the experimental ECD spectrum and the calculated one for **1** showed a negative first Cotton effect at approximately 255 nm and showed the the same trend for other parts (Figure 4). Therefore, qualitative analysis of the calculated and experimental ECD spectra allowed the assignments of the absolute configuration of **1** as 3*R*, 5*S*. The structure of **1** was thus established and named aspidoptoid A as depicted in Figure 1. 

Compound **2** was obtained as a yellow powder with a molecular formula of C_19_H_24_O_3_ on the basis of the positive HRESIMS peak at *m*/*z* 323.1616 [M + Na]^+^ (calcd. for C_19_H_24_O_3_Na, 323.1618), indicating eight degrees of unsaturation and showing 16 mass units more than those of compound **1**. The IR absorptions indicated the presence of hydroxyl (3438 cm^−1^) and olefinic (1631 and 1531cm^−1^) groups. The ^1^H and ^13^C NMR spectral data of **2** (Table 1 and Table 2) showed, in addition to the presence of one terminal vinyl group (*δ*_H_ 6.66, dd, *J* = 18.0, 11.4 Hz, H-15; *δ*_H_ 5.57, dd, *J* = 11.4, 2.0 Hz, H-16*α*; *δ*_H_ 5.19, dd, *J* = 18.0, 2.0 Hz, H-16*β*; *δ*_C_ 135.1 and 120.5) and one penta-substituted benzene ring (*δ*_H_ 6.85, s; *δ*_C_ 152.4, 138.8, 132.7, 130.9, 122.4, and 112.1), easily recognized signals for three singlet methyls, three aliphatic methylenes, three methines (two oxygenated), and two quaternary carbons (one oxygenated and one aliphatic). These data accounted for four out of eight degrees of unsaturations, suggesting that **2** was a tetracyclic compound. A comparison of the ^1^H and ^13^C data of **2** (Table 1 and Table 2) with those of **1** indicated they shared similar structural skeleton and the major change in ring A. Signals for the Δ^1,10^ double bond in **1** were replaced by those for an oxygenated quaternary carbon (*δ*_C_ 87.1) and an oxymethine (*δ*_H_ 4.32, dd, *J* = 10.3, 3.6 Hz; *δ*_C_ 79.9) in **2.** An ether bridge could be present between C-3 and C-10 which was further supported by its HMBC correlation from H-3 (*δ*_H_ 3.86) to C-1/C-5/C-10/C-19 and ^1^H-^1^H COSY correlations H-1/H_2_-2/H-3. The extra hydroxyl group was assigned to C-1 by the HMBC correlations from H-1 (*δ*_H_ 4.32) to C-2 (*δ*_C_ 34.2)/C-3 (*δ*_C_ 86.9)/C-5 (*δ*_C_ 43.8)/C-10 (*δ*_C_ 87.1) and the downfield carbon chemical shift of C-1 (*δ*_C_ 79.9) (Figure 2). The aforementioned information indicated that **2** was a phenylethylene-bearing 20-nor-diterpenoid possessing a rare 3,20-oxybridge.

The ROESY correlations (Figure 3) of H-1/H-2*α*, H-2*α*/H-3, and H-3/H_3_-18 suggested these protons to be α-oriented, while ROESY correlations of H-2*β*/H_3_-19 and H-5/H_3_-19 suggested these protons to be *β*-oriented. The absolute configuration of **2** was finally assigned by comparison of the experimental ECD spectrum with TDDFT calculated spectrum. In the 200–400 nm regions, the calculated spectra showed the same trend as the experimental one, although rotatory strengths of the third positive Cotton effect around 230 nm were overestimated (Figure 4), implying the absolute configuration of **2** as 1*R*, 3*S*, 5*R*, and 10*R.* The structure of **2** was thus established and named aspidoptoid B as shown in Figure 1.

Compound **3**, a yellow powder, possessed a molecular formula of C_18_H_20_O_5_ as deduced from the negative HRESIMS peak at *m*/*z* 315.1234 [M − H]^−^ (calcd. for C_18_H_19_O_5_, 315.1238). The IR spectrum revealed the presence of hydroxyl (3433 cm^−1^) and conjugated carbonyl (1631 cm^−1^). The ^13^C NMR (Table 2) distinguished 18 resonances due to the four methyls (one *O*-methyl), one methylene, four methines (one oxygenated and two olefinic), and nine quaternary carbons (two keto and six olefinic). Analysis of its ^1^H and ^13^C NMR spectra indicated that **3** was a congener of domohinone [10] with the only difference being an OH-14 in **3** instead of an aromatic proton H-14 in domohinone which was confirmed by the HMBC correlations (Figure 2) from OH-14 (*δ*_H_ 12.77) to C-8 (*δ*_C_ 110.5), C-13 (*δ*_C_ 117.6), and C-14 (*δ*_C_ 162.1). 

The relative configuration of **3** was mainly established by a ROESY spectrum. The ROESY correlations (Figure 3) of H-3/H-5, H-3/H_3_-19, H-5/H_3_-19, and H-5/H-6*β* indicated that these protons were all *β*-oriented, whereas the ROESY correlations of H-6*α*/H_3_-18 revealed the *α*-orientation of the corresponding protons. The absolute configurations of **3** were elucidated as 3*S*, 5*S* based on the similar positive Cotton effects at 220 and 300 nm between the experimental ECD spectrum and the calculated spectrum (Figure 4). The structure of **3** was thus established and named aspidoptoid C as depicted in Figure 1.

Compound **4**, a yellow powder, had the molecular formula C_20_H_26_O_3_, as determined by positive HRESIMS peak at *m*/*z* 337.1772 [M + Na]^+^ (calcd. for C_20_H_26_O_3_Na, 337.1774). The IR spectrum exhibited absorption bands for hydroxyl (3428 cm^−1^) and ketone carbonyl (1713 and 1640 cm^−1^). The 1D NMR spectra revealed 20 carbon signals assignable to four methyls, four methylenes (one olefinic), four methines (one oxygenated and two olefinic), and eight quaternary carbons (one keto and six olefinic). Comparing with spruceanol (**5**) [9], the major difference was the presence of a ketone canbonyl (*δ*_C_ 210.7) in **4** instead of a methylene (*δ*_C_ 27.98) in **5**, indicating that **4** was the oxygenated derivative of **5**. The ketone carbonyl was assigned at C-2 by HMBC correlations from H-1 (*δ*_H_ 3.05, d, *J* = 12.3 Hz, H-1α; *δ*_H_ 2.63, d, *J* = 12.3 Hz, H-1*β*), H-3 (*δ*_H_ 3.97, s), and OH-3 (*δ*_H_ 3.47, s) to C-2 (*δ*_C_ 210.7). The planar structure of **4** was further secured by detailed analyses of its 2D NMR data (Figure 2). The ROESY correlations of H-1*β*/H-3, H-3/H-5, H-5/H_3_-19, H-6*β*/H_3_-19, and H-7*β*/H-6*β* showed that they were cofacial and randomly assigned to be β-oriented. Subsequently, the ROESY cross-peaks of H-1*α*/H_3_-20, H-6*α*/H_3_-18, H-6*α*/H_3_-20, and H_3_-18/H_3_-20 indicated that H_3_-18 and H_3_-20 were *α*-oriented (Figure 3). The absolute configuration of **4** was determined by ECD spectrum (Figure 4). The calculated spectra showed the same trend as the experimental one which confirmed the absolute configuration of **4** as 3*S*, 5*S,* and 10*R.* The structure of **4** was established and named aspidoptoid D as depicted in Figure 1.

Two known compounds were identified to be spruceanol (**5**) [9] and sonderianol (**6**) [11] by comparing their spectroscopic data with those in the literature.

**Scheme 1:**
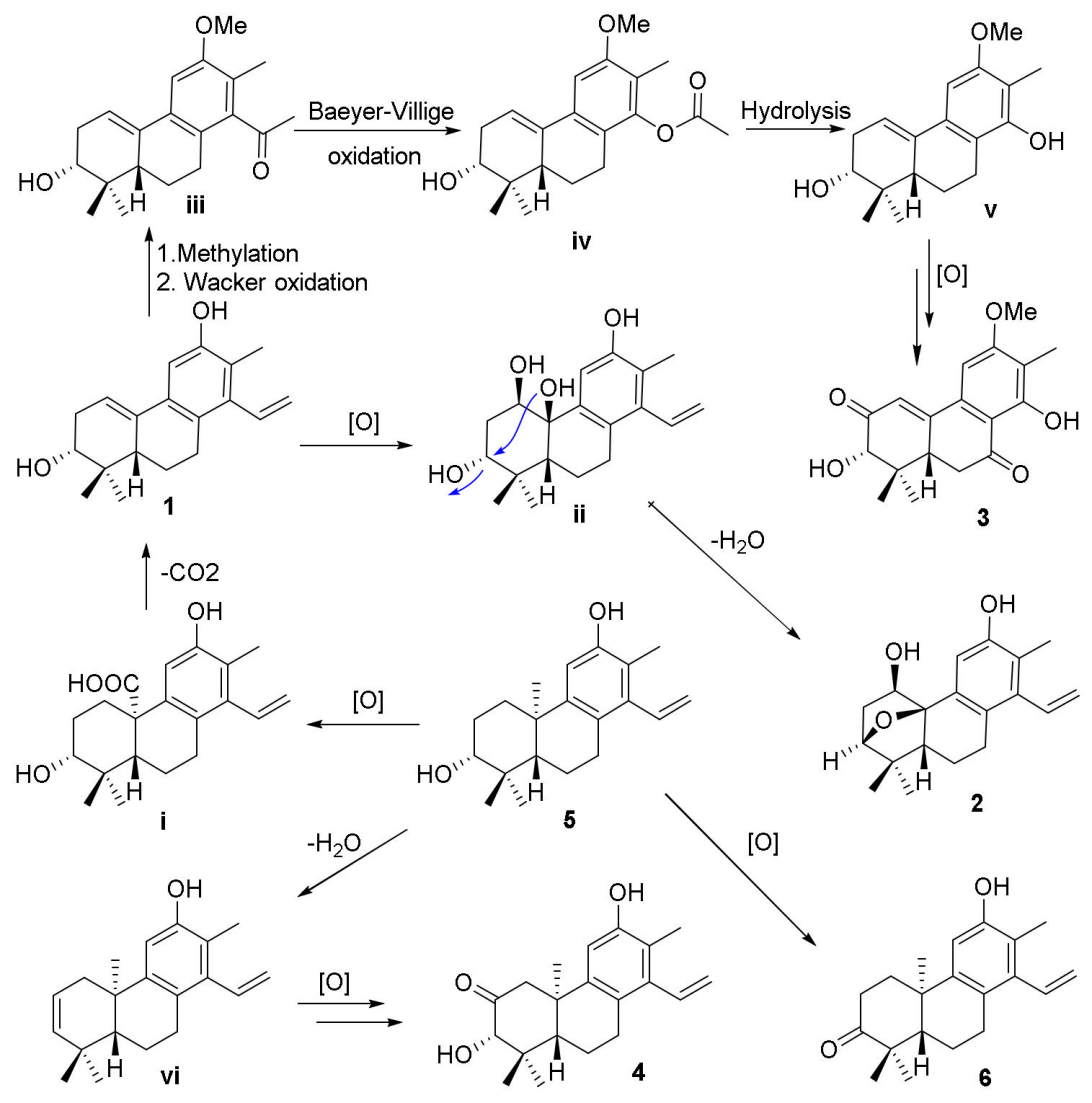
Plausible biosynthetic pathways of compounds **1**–**4**.

The biosynthetic pathways for compounds **1**−**4** are shown in Scheme 1. The precursors for compounds **1**−**4** were considered to be co-isolated cleistanthane-type diterpenoid spruceanol (**5**), which was transformed to intermediate **i** by the oxidation process and then decarboxylation to afford compound **1**. Compound **1** would undergo oxidation and dehydration procedures to give compound **2**. The methylation and Wacker oxidation processes of **1** produced intermediate **iii** which underwent Baeyer−Villiger oxidation and hydrolysis processes to yield intermediate **v**. Compound **3** was finally obtained by the further oxidation reaction of intermediate **v**. Compounds **4** and **6** were also produced after a series of oxidation of compound **5** [12].

All the isolates were evaluated for their inhibitory effects on nitric oxide (NO) production stimulated by LPS in RAW 264.7 cells with l-NMMA (NG-monomethyl l-arginine) as the reference compound. Among those compounds, compound **5** exhibited weak NO inhibition and the rest of the compounds were inactive at 50 μM (Table 3). In addition, they were also tested for their cytotoxic activity against five human tumor cell lines (i.e., HL-60, SMMC-7721, A-549, MCF-7, and SW-480) using the MTS method. However, all the compounds were non-cytotoxic (IC_50_ > 40 μM) (Supplementary Materials Table S1). 

## 3. Materials and Methods 

### 3.1. General Experimental Procedures

Optical rotations were obtained with a Rudolph Autopol VI polarimeter (Rudolph Research Analytical, Hackettstown, NJ, USA). The UV spectra were measured with a Shimadzu UV-2401A instrument (Shimadzu Corporation, Kyoto, Japan). The IR spectra (KBr) were determined on a Bruker Tensor-27 infrared spectrometer (Bruker Corporation, Karlsruhe, Germany). The ECD spectra were measured with a Chirascan circular dichroism spectrometer (Applied Photophysics Ltd., Surrey, UK). The NMR spectra were obtained on a Bruker Avance spectrometer operating (Bruker Corporation, Karlsruhe, Germany) at 600 MHz for ^1^H NMR and 150 MHz for ^13^C NMR, using tetramethylsilane as an internal standard. The ESIMS and HRESIMS were carried out on a Shimadzu UPLC-IT-TOF mass spectrometer (Shimadzu Corporation, Kyoto, Japan). Semi-preparative HPLC was performed on a Waters 600 pump system with a 2996 photodiode array detector using a YMC-Pack ODS-A column (300 × 10 mm, S-5 μM, YMC Co., Ltd., Komatsu, Japan). The TLC and column chromatography (CC) were performed on plates precoated with silica gel GF254 (10−40 μm) and over silica gel (200−300 mesh, Qingdao Marine Chemical Factory, Qingdao, China), Sephadex LH-20 gel (40−70 μM, Amersham Pharmacia Biotech AB, Uppsala, Sweden), C18 reversed-phase silica gel (40-63 μm; Merck, Darmstadt, Germany), and MCI gel (CHP20/P120, 75−150 μm, high-porous polymer, Mitsubishi Chemical Corporation, Tokyo, Japan). All solvents used were of analytical grade (Shanghai Chemical Reagents Co. Ltd., Shanghai, China), and all solvents used for the HPLC were of spectral grade (J & K Scientific Ltd., Beijing, China).

### 3.2. Plant Material

The vine of *A. obcordata* was collected from Xishuangbanna Tropical Botanical Garden (XTBG), Chinese Academy of Science (CAS), Mengla Country, Yunnan Province, People’s Republic of China, in September 2016, and they were identified by one of the authors (C.-F.X.). A voucher specimen (No. HITBC-094469) was deposited in the herbarium of XTBG.

### 3.3. Extraction and Isolation

Air-dried powder of *A. obcordata* vine (5.5 kg) was extracted three times with 95% EtOH (20 L, *v*/*v*) (one week for each time) at room temperature. The extracts were combined and concentrated under reduced pressure. Then, the concentrate (480.0 g) was suspended in H_2_O (2 L) and successively partitioned with ethyl acetate and *n*-BuOH. The ethyl acetate fraction (150.0 g) was separated over a MCI gel column (8 cm × 100 cm) chromatograph and eluted with EtOH/H_2_O (40/60 to 90/10, *v*/*v*, each 8 L) to obtain fractions 1 to 6. Fraction 6 (33.2 g) was then subjected to silica gel CC (6 cm × 70 cm, 200–300 mesh) and eluted with a gradient of CH_2_Cl_2_/MeOH (1:0 to 1:1, *v*/*v*, each 4 L) to produce fractions A–E (8.5, 5.0, 10.4, 2.7, and 3.2 g, respectively). Fraction B (5.0 g) was subjected to silica gel CC (4 cm × 70 cm, 200–300 mesh) and eluted with a gradient of CH_2_Cl_2_/MeOH (1:0 to 20:1, *v*/*v*, each 1 L) to produce fractions B1–B3. Fraction B1 (1.2 g) was further purified by semi-preparative HPLC (10 mm × 300 mm, MeCN/H_2_O, 90:10, *v*/*v*, 3 mL/min) to yield **1** (5 mg), **5** (10 mg), and **2** (4 mg), respectively. Fraction C (10.4 g) was further separated by CC over a Sephadex LH-20 (2 cm × 100 cm) column (MeOH as eluent) to give three major subfractions, purification of which by semi-preparative HPLC (10 mm × 300 mm, MeCN/H_2_O, 82:18, *v*/*v*, 3 mL/min) yielded **3** (12 mg), **6** (8 mg), and **4** (7 mg), respectively. 

*Aspidoptoid A* (**1**): Yellow powder; [α]^25.2^_d_ −27.1° (*c* 0.16, MeOH); UV (MeOH) *λ*_max_ (log ε) 232 (3.8), 259 (3.5), 314 (3.1) nm; IR (KBr) *ν*_max_ 3431, 2964, 2927, 2870, 1708, 1630, 1404, 1384, 1364, 1322, 1293, 1117, 995, 963, 923, 895, 857, 833 cm^−1^; negative HRESIMS *m*/*z* 283.1704 [M − H]^−^ (calcd. for C_19_H_23_O_2_, 283.1704); for ^1^H and ^13^C NMR data, see Table 1 and Table 2.

*Aspidoptoid B* (**2**): Yellow powder; [α]^24.9^_D_ −20.5° (*c* 0.07, MeOH); UV (MeOH) *λ*_max_ (log ε) 205 (3.2), 268 (2.3) nm; IR (KBr) *ν*_max_ 3438, 2961, 2925, 2855, 1722, 1705, 1631, 1550, 1531, 1463, 1408, 1384, 1262, 1097, 1023, 872, 804, 559 cm^−1^; positive HRESIMS *m*/*z* 323.1616 [M + Na]^+^ (calcd. for C_19_H_24_O_3_Na, 323.1618); for ^1^H and ^13^C NMR data, see Table 1 and Table 2.

*Aspidoptoid C* (**3**): Yellow powder; [α]^25.1^_D_ −14.2° (*c* 0.13, MeOH); UV (MeOH) *λ*_max_ (log ε) 205 (3.2), 269 (3.3), 329 (3.0), 356 (2.8) nm; IR (KBr) *ν*_max_ 3433, 2959, 2925, 2854, 1631, 1220, 1191, 1163, 1143, 1128, cm^−1^; negative HRESIMS *m*/*z* 315.1234 [M − H]^−^ (calcd. for C_18_H_19_O_5_, 315.1238); for ^1^H and ^13^C NMR data, see Table 1 and Table 2.

*Aspidoptoid D* (**4**): Yellow powder; [α]^24.7^_D_ −9.7° (*c* 0.15, MeOH); UV (MeOH) *λ*_max_ (log ε) 205 (3.8), 238 (3.3), 298 (3.0), 354 (2.7) nm; IR (KBr) *ν*_max_ 3428, 3084, 2968, 2926, 2872, 2854, 1713, 1640, 1592, 1511, 1466, 1162, 1001, 965, 923, 856, 803, 685, 621 cm^−1^; positive HRESIMS *m*/*z* 337.1772 [M + Na]^+^ (calcd. for C_20_H_26_O_3_Na, 337.1774); for ^1^H and ^13^C NMR data, see Table 1 and Table 2.

### 3.4. Assay for Inhibition Ability toward LPS-Induced NO Production and Cytotoxicity Testing

The RAW 264.7 macrophages (obtained from Kunming Institute of Zoology, Chinese Academy of Sciences) were maintained in DEMEM/high-glucose medium (Invitrogen, Carlsbad, CA, USA) supplemented with 10% (*v*/*v*) newborn calf serum and antibiotics (100 U/mL penicillin and 0.1 g/L streptomycin) at 37 °C in the presence of 5% CO_2_. The cell viability was determined by MTS assay before the nitric oxide (NO) production assay, and the NO production was measured by the accumulation of nitrite in the culture supernatants using the Griess Reagent System as previously reported [13]. All experiments were performed in three independent replicates, and l-NMMA (NG-monomethyl L-arginine) (Sigma) was used as a positive control. Statistical analysis was calculated using SPSS 21.0 software (International Business Machines Corporation, Armonk, NY, USA).

### 3.5. Cytotoxicity Assay

Cytotoxic activity of the compounds against the HL-60, SMMC-7721, A-549, MCF-7, and SW480 cell lines were evaluated using the MTS method [14]. Briefly, all cells were cultured in RPMI 1640 medium containing 10% fetal bovine serum and 100 U/mL penicillin/streptomycin in a humidified incubator in a 5% CO_2_ atmosphere at 37 °C. Then, 100 μL of adherent cells was seeded into each well (1 × 10^4^ cells/well) of 96 well cell culture plates and allowed to adhere for 12 h before test drug addition. Each tumor cell line was exposed to a test compound at concentrations of 0.064, 0.32, 1.6, 8, and 40 μM in DMSO in triplicate for 48 h, with cisplatin as the positive control. After 48 h incubation, 20 μL of MTS [3-(4,5-dimethylthiazol-2-yl)-5-(3-carboxymethoxy-phenyl)-2-(4-sulfophenyl)-2*H*- tetrazolium] solution was added to each well which were incubated for another 4 h to give a formazan product. Then 100 μL of 20% SDS was added to each well and incubated 12 h at room temperature for the formazan product to dissolve completely. The OD value of each well was measured at 490 nm using a Biorad 680 instrument (Bio-Rad Laboratories, Inc., Hercules, CA, USA). The IC_50_ value of each compound was calculated by the Reed and Muench method [15].

## 4. Conclusions

In summary, four new ones aspidoptoids A–D (**1**–**4**) together with two known analogues were isolated and characterized by solid data from *A. obcordata* vine. Compounds **1** and **2** are the first examples of phenylethylene-bearing 20-nor-diterpenoids, and **2** possesses a rare 3,10-oxybridge. The plausible biosynthetic pathways for compounds **1**–**4** were also proposed with compound **5** as the precursor. All the isolates were evaluated for their cytotoxicity and NO inhibitory effects. Among those compounds, compound **5** exhibited weak NO inhibitory effects at 50 μM.

## Figures and Tables

**Table 1:** ^1^H (600 MHz) NMR data for compounds **1**–**4** in CDCl_3_.

No.	1	2	3	4
	*δ*_H_, mult. (*J* in Hz)	*δ*_H_, mult. (*J* in Hz)	*δ*_H_, mult. (*J* in Hz)	*δ*_H_, mult. (*J* in Hz)
1*α*	6.18, t (3.1)	4.32, dd (10.3, 3.6)	6.83, d (2.7)	3.05, d (12.3)
1*β*				2.63, d (12.3)
2*α*	2.16, ddd (10.2, 6.5, 3.1)	1.81, dd (13.2, 3.6)		
2*β*	2.47, m	2.30, m		
3	3.60, overlapped	3.86, d (5.9)	4.09, s	3.97, s
5	2.02, m	2.38, dd (13.6, 4.8)	3.15, ddd (13.5, 5.2, 2.7)	1.96, dd (12.2, 2.0)
6*α*	1.37, ddd (26.3, 7.2, 2.9)	1.28, m	2.73, dd (16.4, 13.5)	1.75, ddd (25.2, 12.2, 6.0)
6*β*	2.03, m	1.78, m	2.88, dd (16.4, 5.2)	2.01, m
7*α*	2.87, ddd (16.5, 4.1, 2.9)	2.88, m		2.84, dd (17.3, 6.0)
7*β*	2.5, m	2.30, m		2.67, m
11	7.03, s	6.85, s	6.80, s	6.54, s
15	6.61, dd (17.9, 11.4)	6.66, dd (18.0, 11.4)		6.58, dd (18.0, 11.4)
16*α*	5.55, dd (11.4, 2.0)	5.57, dd (11.4, 2.0)		5.56, dd (11.4, 2.0)
16*β*	5.17, dd (17.9, 2.0)	5.19, dd (18.0, 2.0)		5.18, dd (18.0, 2.0)
17	2.19, s	2.19, s	2.13, s	2.19, s
18	0.77, s	1.02, s	0.85, s	0.78, s
19	1.11, s	1.20, s	1.29, s	1.25, s
20				1.18, s
OMe-12			3.93, s	
OH-3				3.47, s
OH-14			12.77, s	

**Table 2:** ^13^C (150 MHz) NMR data for compounds **1**–**4** in CDCl_3_.

No.	1	2	3	4
	*δ*_C_	*δ*_C_	*δ*_C_	*δ*_C_
1	116.5	79.9	119.6	52.1
2	32.3	34.2	199.1	210.7
3	74.9	86.9	79.7	82.8
4	37.2	44.6	41.3	44.9
5	46.2	43.8	46.0	48.8
6	24.0	22.7	37.4	19.2
7	28.9	28.3	201.4	29.1
8	127.3	122.4	110.5	125.1
9	132.9	130.9	136.0	145.4
10	134.5	87.1	153.6	43.8
11	108.3	112.1	99.8	109.4
12	152.1	152.4	163.6	152.5
13	121.3	132.7	117.6	120.2
14	139.5	138.8	162.1	139.7
15	135.3	135.1		135.3
16	120.1	120.5		120.1
17	13.1	13.0	8.1	13.0
18	13.4	24.2	14.1	16.4
19	24.4	24.5	24.4	29.3
20				26.1
OMe-12			56.0	

**Table 3:** NO inhibitory effects of compounds **1**–**6**.

Compound	Concentration (μΜ)	NO Inhibition Rate (%)
**1**	50	−5.94 ± 2.27
**2**	50	−0.56 ± 1.09
**3**	50	−3.50 ± 1.60
**4**	50	−7.78 ± 1.50
**5**	50	31.54 ± 1.55
**6**	50	−11.45 ± 1.93
l-NMMA ^a^	50	51.23 ± 0.65

^a^ Positive control.

## Supplementary Materials

The following are available online, Figure S1–S41: 1D and 2D NMR, HRESIMS, UV, IR spectra, ECD data of new compounds **1**–**4**. Table S1: Cytotoxic activity of compounds **1**–**6**
