# Supplementary material for: Aspidoptoids A–D: Four New Diterpenoids from Aspidopterys obcordata Vine
Source: Molecules. 2020 Jan 25;25(3):529. doi: 10.3390/molecules25030529 (PMC7036900; doi:10.3390/molecules25030529)
Supplement: Supplementary file 1 [file molecules-25-00529-s001.pdf]

Aspidoptoids A–D: Four New Diterpenoids from *Aspidopterys obcordata* Vine

Peng Sun [1,2,†], Dong-Hua Cao [1,2,†], Yi-Dian Xiao [3,4], Zong-Yi Zhang [1,2], Jia-Nan Wang [1,2], Xiao-Cui Shi [1,2], Chun-Fen Xiao [1], Hua-Bin Hu [1] and You-Kai Xu [1,*]

[1] CAS Key Laboratory of Tropical Plant Resources and Sustainable Use, Xishuangbanna Tropical Botanical Garden, Chinese Academy of Sciences, Menglun 666303, China; sunpeng@xtbg.ac.cn (P.S.); caodonghua@xtbg.ac.cn (D.-H.C.); zhangzongyi@xtbg.ac.cn (Z.-Y.Z.); wangjianan@xtbg.ac.cn (J.-N.W.); shixiaocui@xtbg.ac.cn (X.-C.S.); xiaocf@xtbg.ac.cn (C.-F.X.); huhb@xtbg.ac.cn (H.-B.H.); xyk@xtbg.ac.cn (Y.-K.X.)

[2] University of Chinese Academy of Sciences, Beijing 100049, China

[3] School of Chemical Science and Technology, Key Laboratory of Medicinal Chemistry for Nature Resources, Ministry of Education, Yunnan University, Kunming 650019, China; xiaoyidian@mail.kib.ac.cn

[4] State Key Laboratory of Phytochemistry and Plant Resources in West China, Kunming Institute of Botany, Chinese Academy of Sciences, Kunming 650201, China

* Correspondence: xyk@xtbg.ac.cn; Tel.: +86 691 871 5071

† These authors contributed equally to this work.

Content of Supporting Information

No.	contents	page
1	**Table S1** Cytotoxic activity of compounds **1-6** (IC_{50}, μM)	4
2	**Figure S1** ^{1}H NMR (600 MHz) spectrum of aspidoptoid A (**1**) in $CDCl_3$	4
3	**Figure S2** ^{13}C NMR (150 MHz) spectrum of aspidoptoid A (**1**) in $CDCl_3$	5
4	**Figure S3** HSQC spectrum of aspidoptoid A (**1**) in $CDCl_3$	5
5	**Figure S4** ^{1}H-^{1}H COSY Spectrum of aspidoptoid A (**1**) in $CDCl_3$	6
6	**Figure S5** HMBC spectrum of aspidoptoid A (**1**) in $CDCl_3$	6
7	**Figure S6** ROESY spectrum of aspidoptoid A (**1**) in $CDCl_3$	7
8	**Figure S7** Negative HR-ESI-MS of aspidoptoid A (**1**)	8
9	**Figure S8** IR spectrum of aspidoptoid A (**1**)	9
10	**Figure S9** UV spectrum of aspidoptoid A (**1**)	9
11	**Figure S10** ^{1}H NMR (600 MHz) spectrum of aspidoptoid B (**2**) in $CDCl_3$	10
12	**Figure S11** ^{13}C NMR (150 MHz) spectrum of aspidoptoid B (**2**) in $CDCl_3$	10
13	**Figure S12** HSQC spectrum of aspidoptoid B (**2**) in $CDCl_3$	11
14	**Figure S13** ^{1}H-^{1}H COSY Spectrum of aspidoptoid B (**2**) in $CDCl_3$	11
15	**Figure S14** HMBC spectrum of aspidoptoid B (**2**) in $CDCl_3$	12
16	**Figure S15** ROESY spectrum of aspidoptoid B (**2**) in $CDCl_3$	12
17	**Figure S16** Postitive HR-ESI-MS of aspidoptoid B (**2**)	13
18	**Figure S17** IR spectrum of aspidoptoid B (**2**)	14
19	**Figure S18** UV spectrum of aspidoptoid B (**2**)	14
20	**Figure S19** ^{1}H NMR (600 MHz) spectrum of aspidoptoid C (**3**) in $CDCl_3$	15
21	**Figure S20** ^{13}C NMR (150 MHz) spectrum of aspidoptoid C (**3**) in $CDCl_3$	15
22	**Figure S21** HSQC spectrum of aspidoptoid C (**3**) in $CDCl_3$	16
23	**Figure S22** ^{1}H-^{1}H COSY Spectrum of aspidoptoid C (**3**) in $CDCl_3$	16
24	**Figure S23** HMBC spectrum of aspidoptoid C (**3**) in $CDCl_3$	17
25	**Figure S24** ROESY spectrum of aspidoptoid C (**3**) in $CDCl_3$	17
26	**Figure S25** Negitive HR-ESI-MS of aspidoptoid C (**3**)	18
27	**Figure S26** IR spectrum of aspidoptoid C (**3**)	19
28	**Figure S27** UV spectrum of aspidoptoid C (**3**)	19
29	**Figure S28** ^{1}H NMR (600 MHz) spectrum of aspidoptoid D (**4**) in $CDCl_3$	20
30	**Figure S29** ^{13}C NMR (150 MHz) spectrum of aspidoptoid D (**4**) in $CDCl_3$	20
31	**Figure S30** HSQC spectrum of aspidoptoid D (**4**) in $CDCl_3$	21
32	**Figure S31** ^{1}H-^{1}H COSY Spectrum of aspidoptoid D (**4**) in $CDCl_3$	21
33	**Figure S32** HMBC spectrum of aspidoptoid D(**4**) in $CDCl_3$	22
34	**Figure S33** ROESY spectrum of aspidoptoid D (**4**) in $CDCl_3$	22
35	**Figure S34** Postitive HR-ESI-MS of aspidoptoid D (**4**)	23
36	**Figure S35** IR spectrum of aspidoptoid D (**4**)	24
37	**Figure S36** UV spectrum of aspidoptoid D (**4**)	24

38	**Figure S37** Experimental (black line) and B3LYP/6-311+G(2d,2p) //B3LYP/6-31G(d) calculated ECD spectra of **1-4** (red line)	25
39	**Figure S38** B3LYP/6-311+G(d) optimized lowest energy 3D conformers of **1**	26
40	**Figure S39** B3LYP/6-311+G(d) optimized lowest energy 3D conformers of **2**	27
41	**Figure S40** B3LYP/6-311+G(d) optimized lowest energy 3D conformers of **3**	28
42	**Figure S41** B3LYP/6-311+G(d) optimized lowest energy 3D conformers of **4**	29

Table S1. Cytotoxic activity of compounds **1-6** (IC_{50}, μM).

Compound	HL-60	SMMC-7721	A-549	MCF-7	SW480
**1**	>40	>40	>40	>40	>40
**2**	>40	>40	>40	>40	>40
**3**	>40	>40	>40	>40	>40
**4**	>40	>40	>40	>40	>40
**5**	>40	>40	>40	>40	>40
**6**	>40	>40	>40	>40	>40
Cisplatin[a]	4.1	15.2	22	34.6	30.6

[a] Positive control.

Figure S1 ^1H NMR (600 MHz) spectrum of aspidoptoid A (**1**) in $CDCl_3$

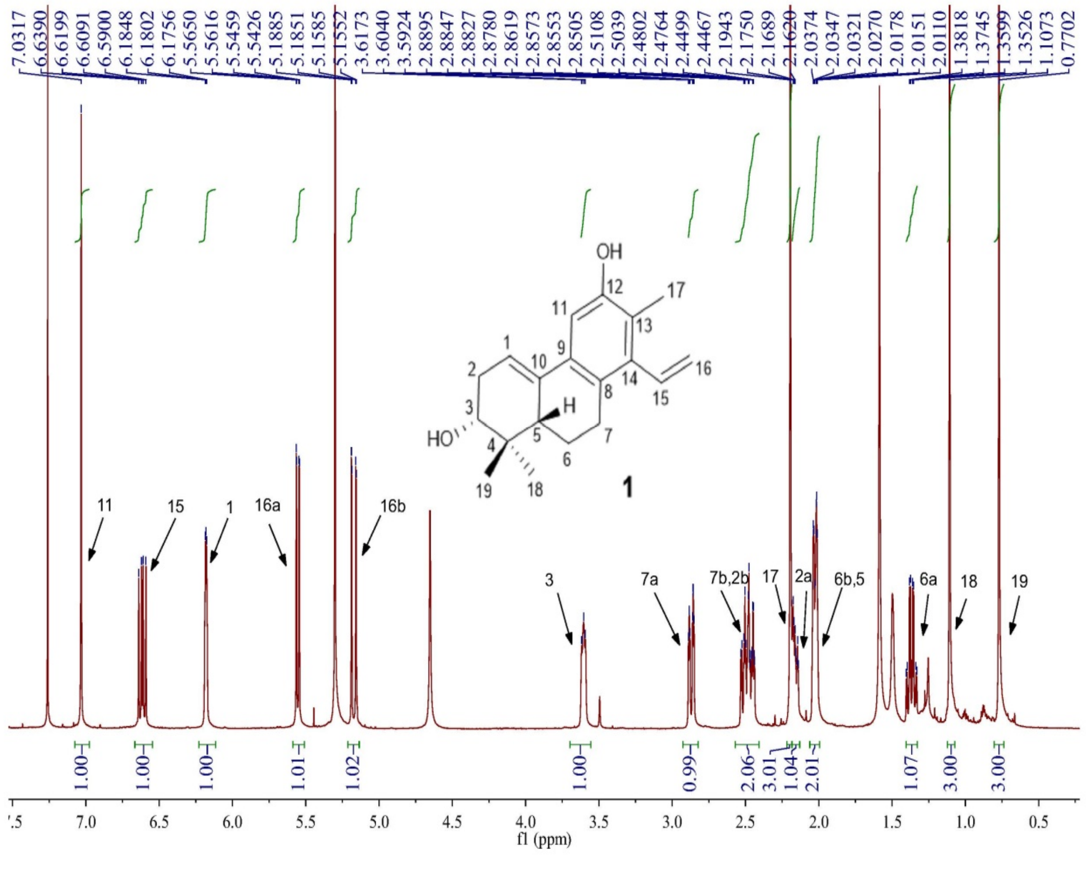

Figure S2 ^{13}C NMR (150 MHz) spectrum of aspidoptoid A (**1**) in $CDCl_3$

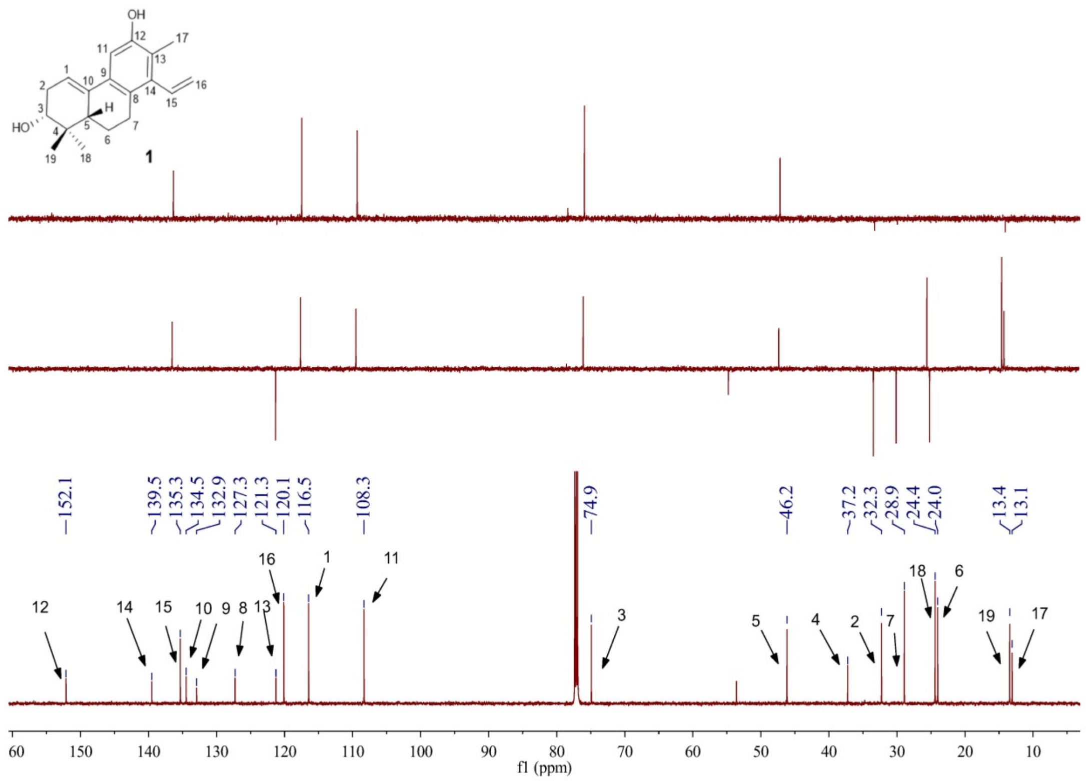

Figure S3 HSQC spectrum of aspidoptoid A (**1**) in $CDCl_3$

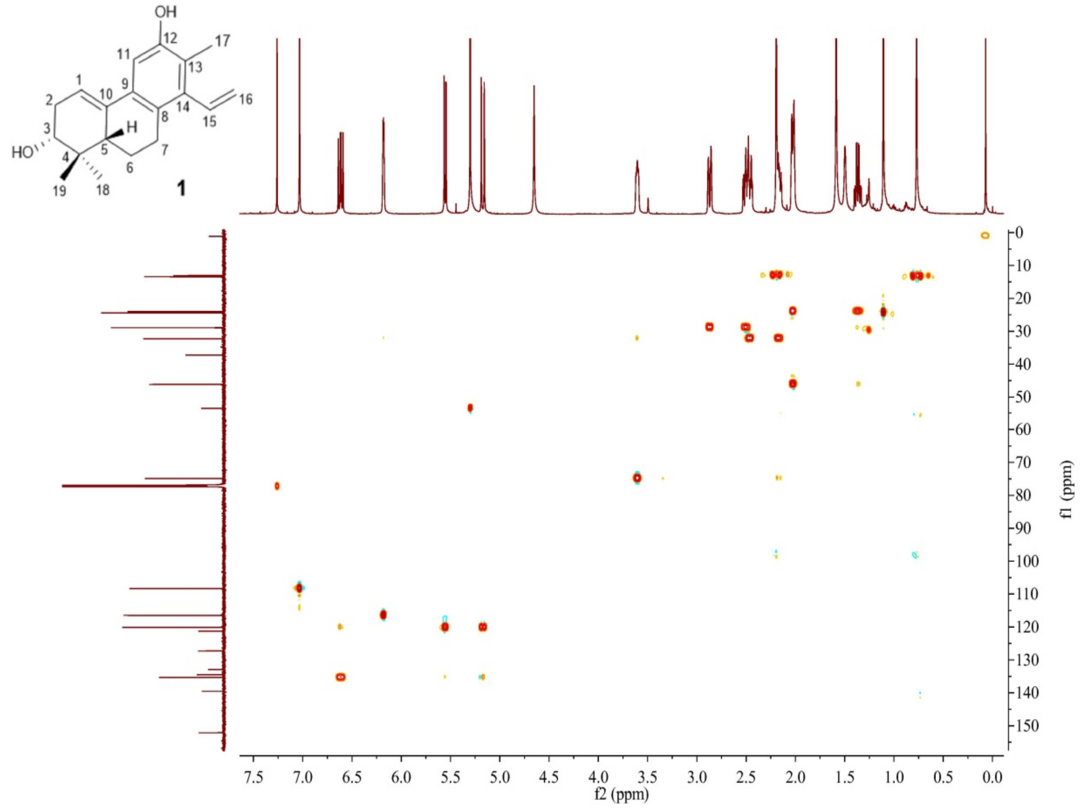

Figure S4 ^{1}H-^{1}H COSY Spectrum of aspidoptoid A (**1**) in $CDCl_3$

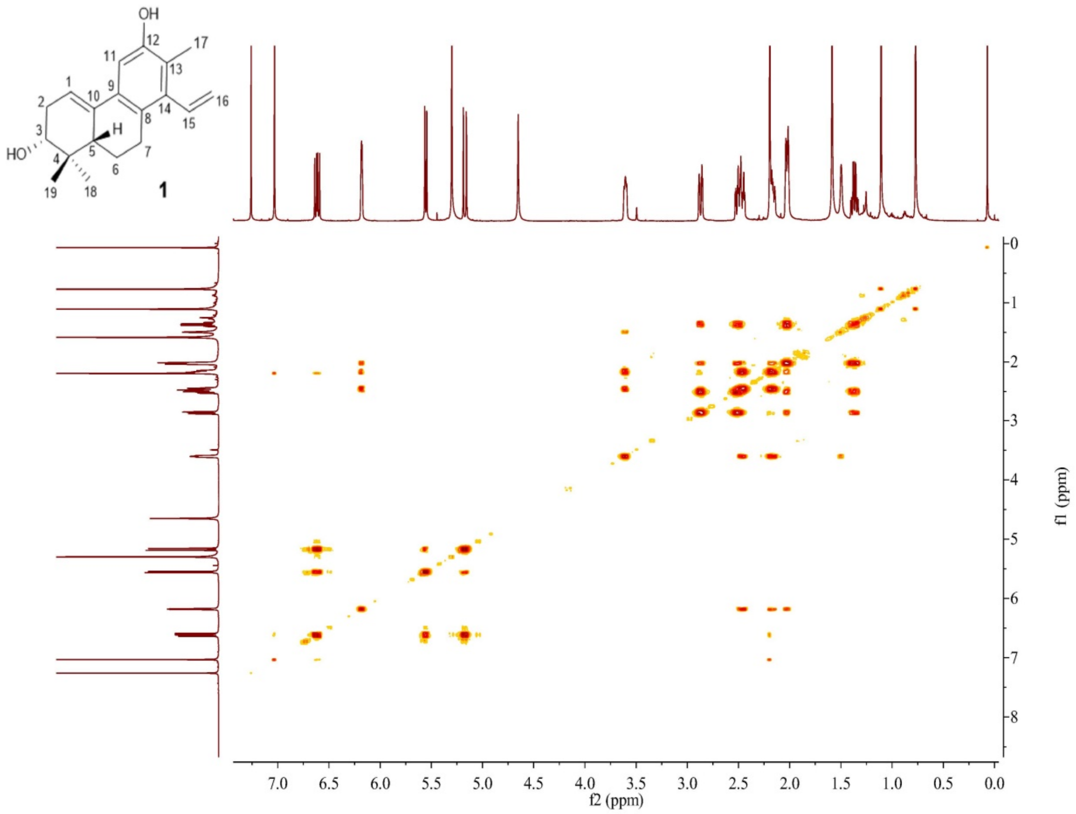

Figure S5 HMBC spectrum of aspidoptoid A (**1**) in $CDCl_3$

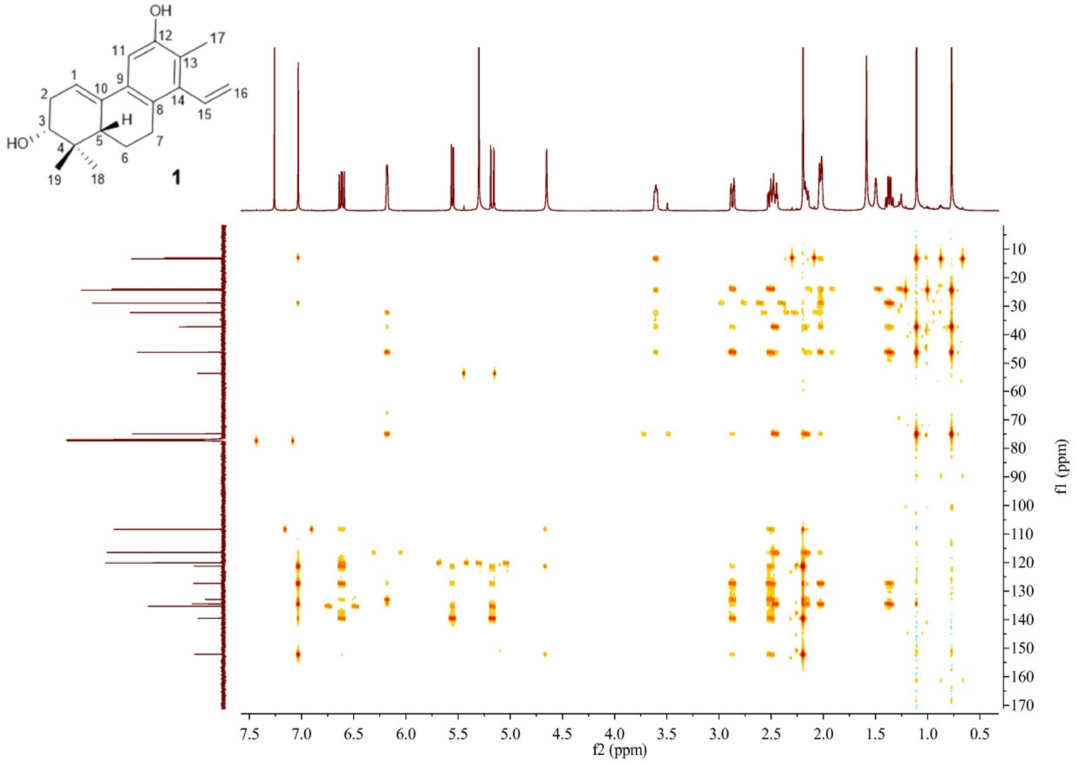

Figure S6 ROESY spectrum of aspidoptoid A (**1**) in $CDCl_3$

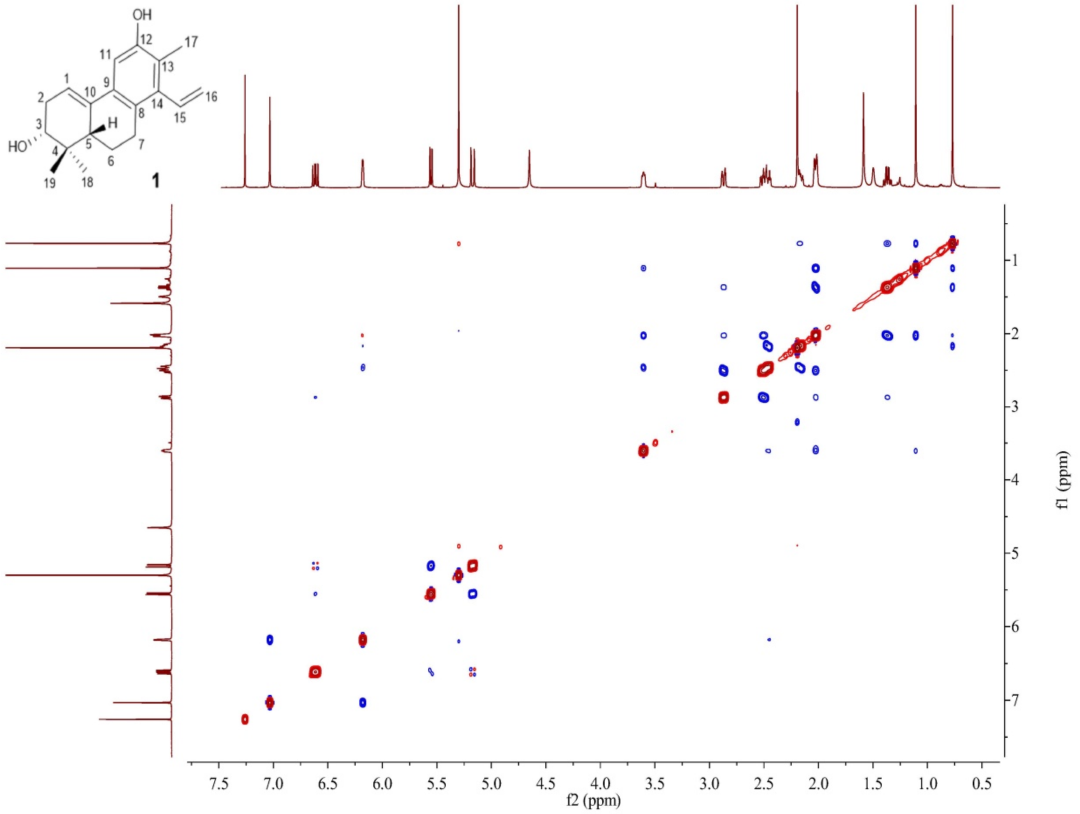

Figure S7 Negative HR-ESI-MS of aspidoptoid A (**1**)

Formula Predictor Report - sd14.lcd

Data File: E:\DATA\2018\0827\sd14.lcd

Elmt	Val.	Min	Max	Elmt	Val.	Min	Max	Elmt	Val.	Min	Max	Elmt	Val.	Min	Max	Use Adduct
H	1	1	100	O	2	0	10	Si	4	0	0	Br	1	0	0	H
C	4	10	50	F	1	0	0	S	2	0	0	I	3	0	0	
N	3	0	0	Na	1	0	0	Cl	1	0	0					

Error Margin (ppm):	5	DBE Range:	-2.0 - 100.0	Electron Ions:	both
HC Ratio:	unlimited	Apply N Rule:	yes	Use MSn Info:	yes
Max Isotopes:	all	Isotope RI (%):	1.00	Isotope Res:	10000
MSn Iso RI (%):	75.00	MSn Logic Mode:	AND	Max Results:	10

Event#: 2 MS(E-) Ret. Time : 0.587 Scan# : 90

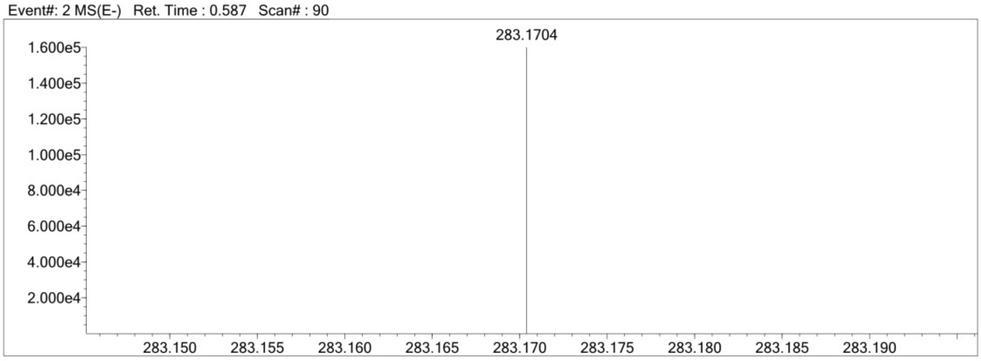

Measured region for 283.1704 m/z

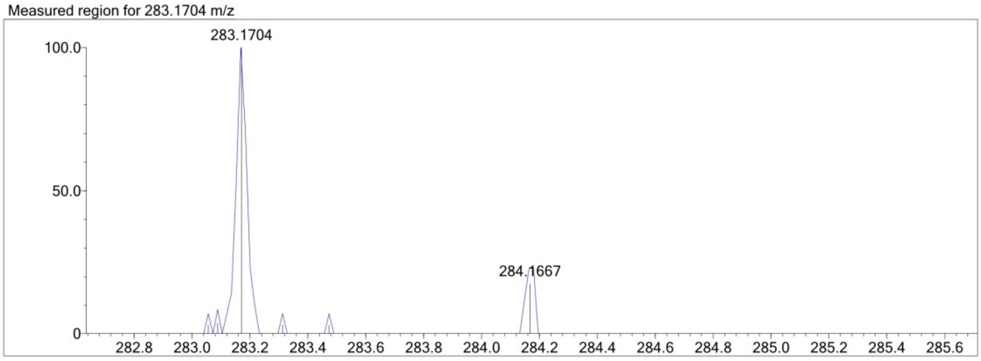

C19 H24 O2 [M-H]- : Predicted region for 283.1704 m/z

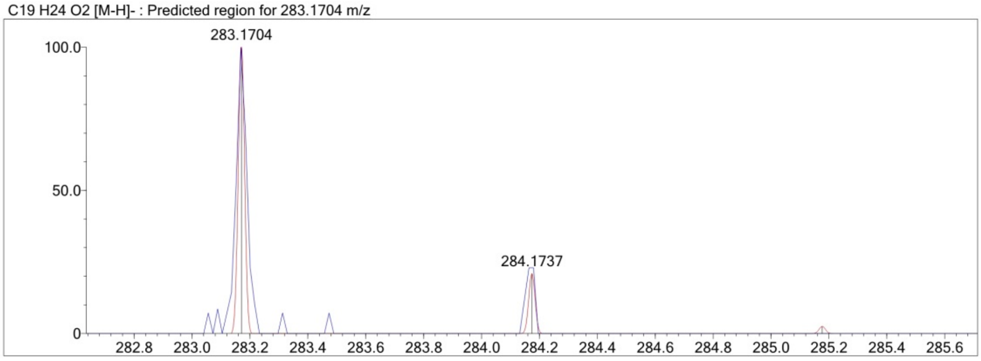

Formula (M)	Ion	Meas. m/z	Pred. m/z	Df. (mDa)	Df. (ppm)	DBE
C19 H24 O2	[M-H]-	283.1704	283.1704	0.0	0.00	8.0

Figure S8 IR spectrum of aspidoptoid A (**1**)

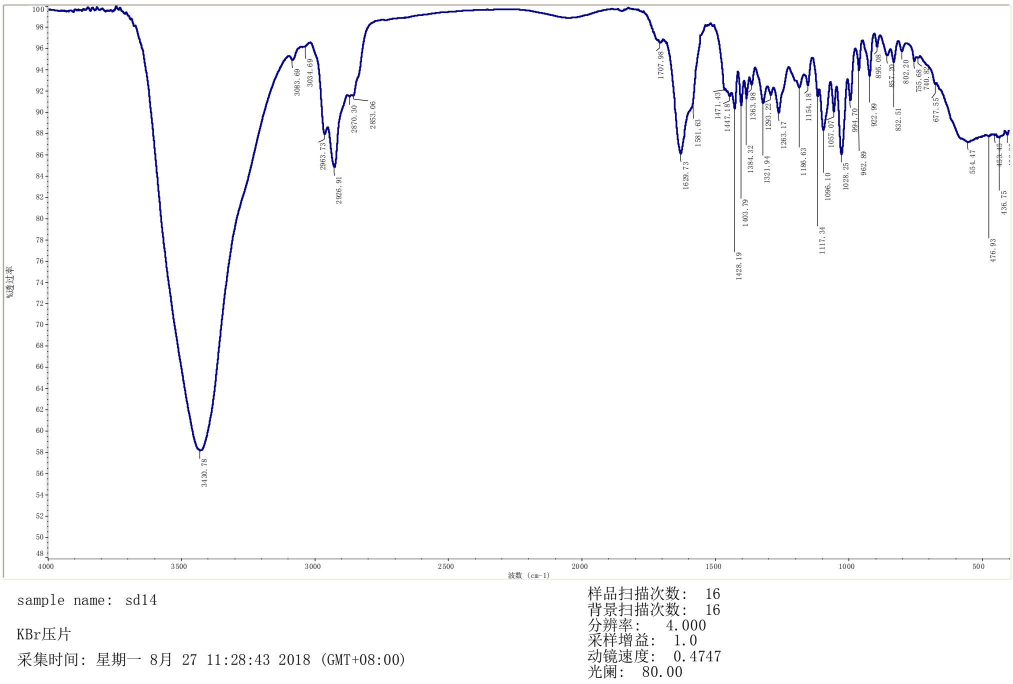

Figure S9 UV spectrum of aspidoptoid A (**1**)

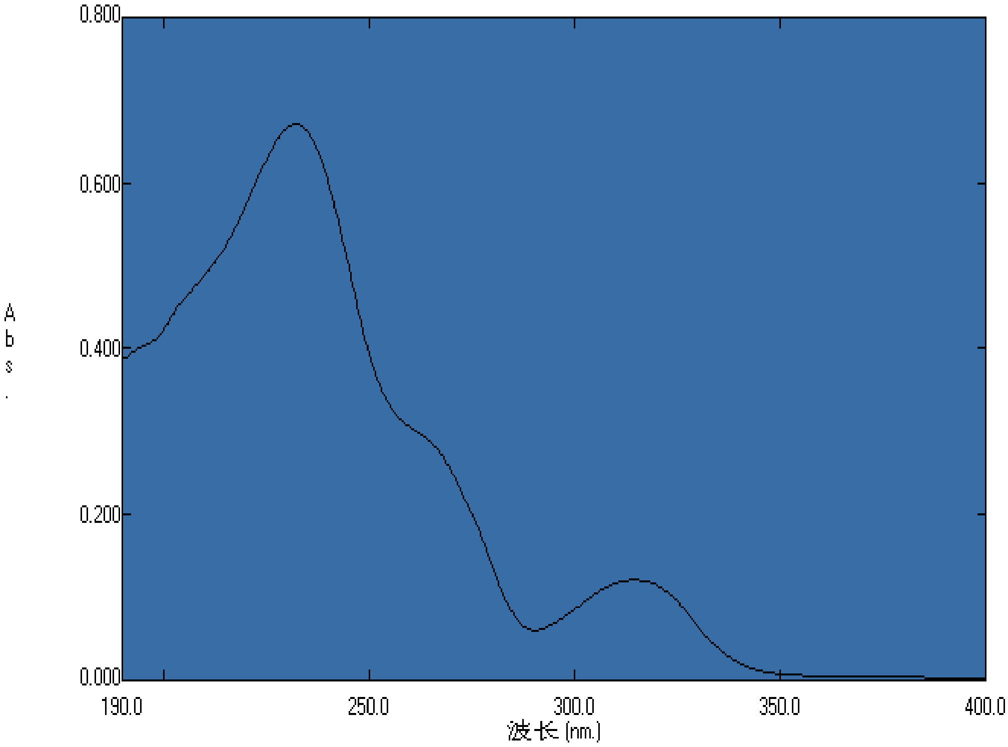

Figure S10 ^{1}H NMR (600 M) spectrum of aspidoptoid B (**2**) in $CDCl_3$

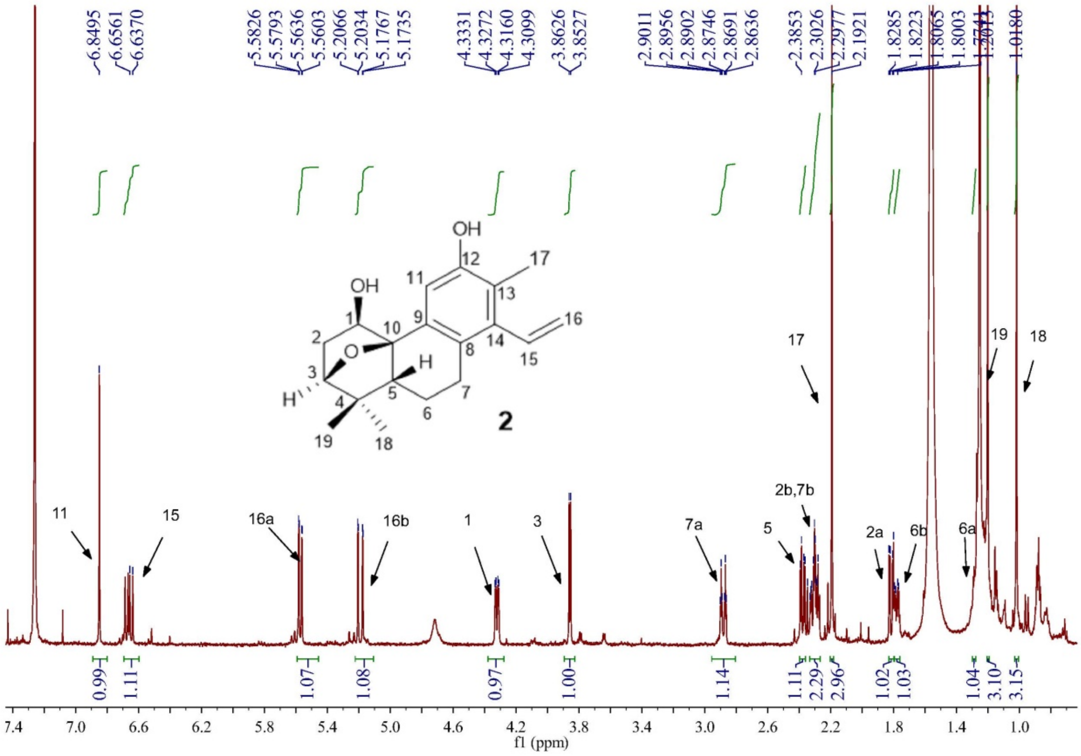

Figure S11 ^{13}C NMR (150 M) spectrum of aspidoptoid B (**2**) in $CDCl_3$

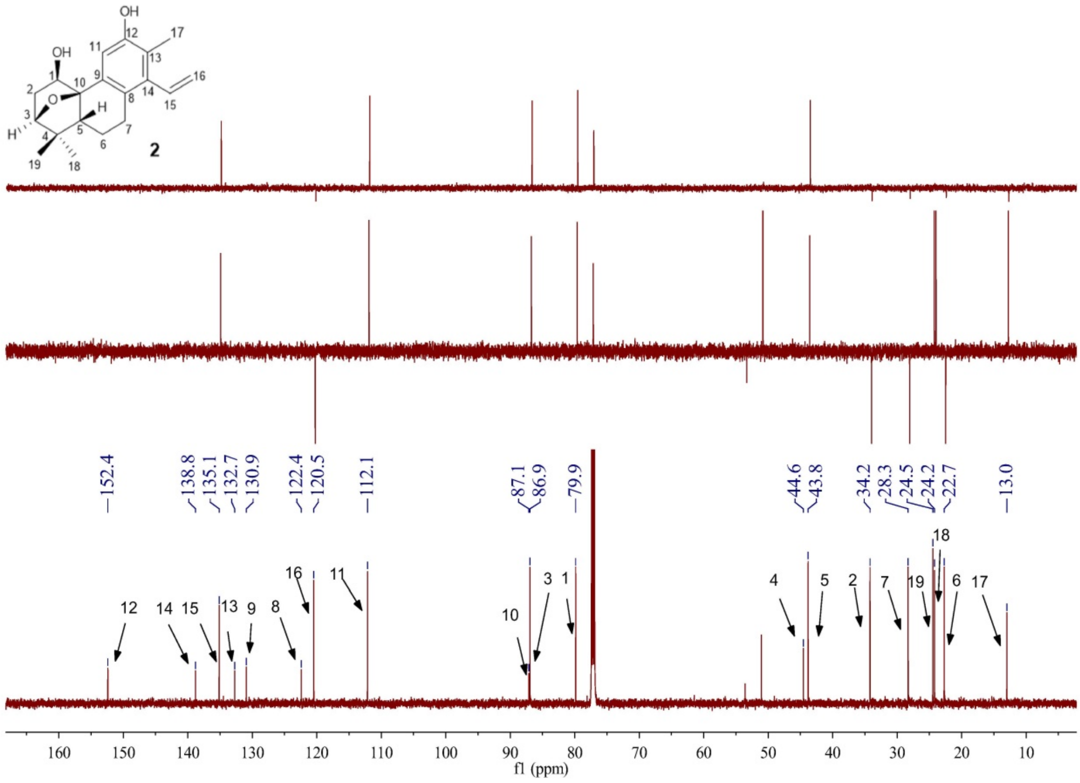

Figure S12 HSQC spectrum of aspidoptoid B (**2**) in $CDCl_3$

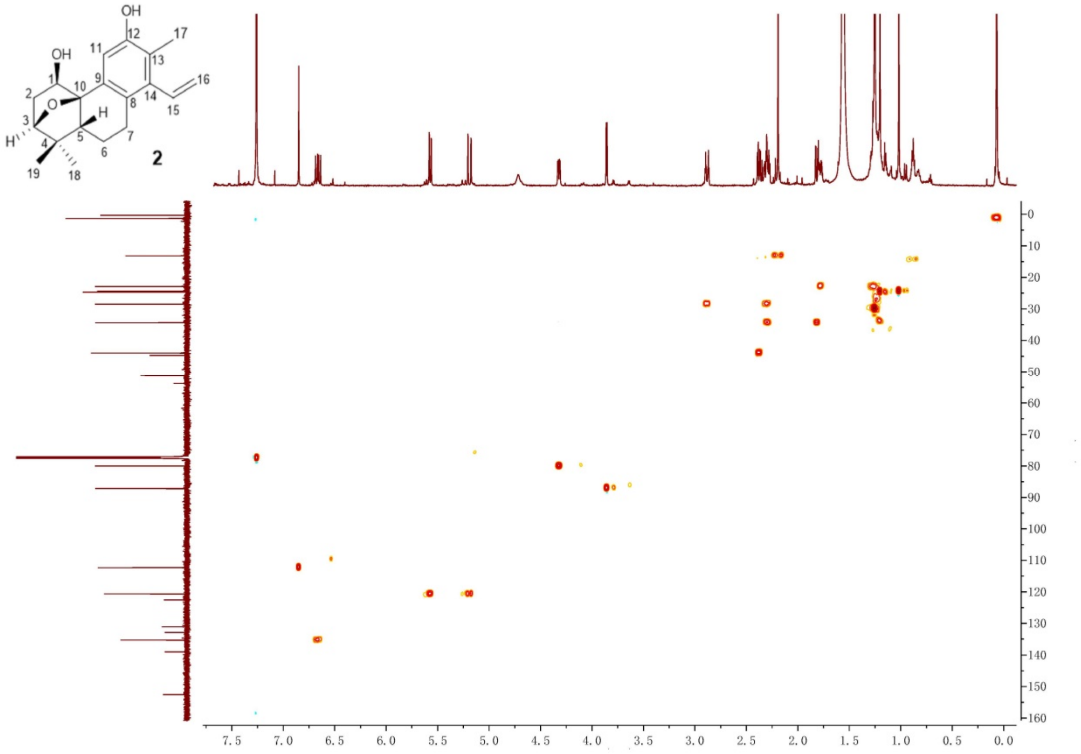

Figure S13 ^1H-^1H COSY spectrum of aspidoptoid B (**2**) in $CDCl_3$

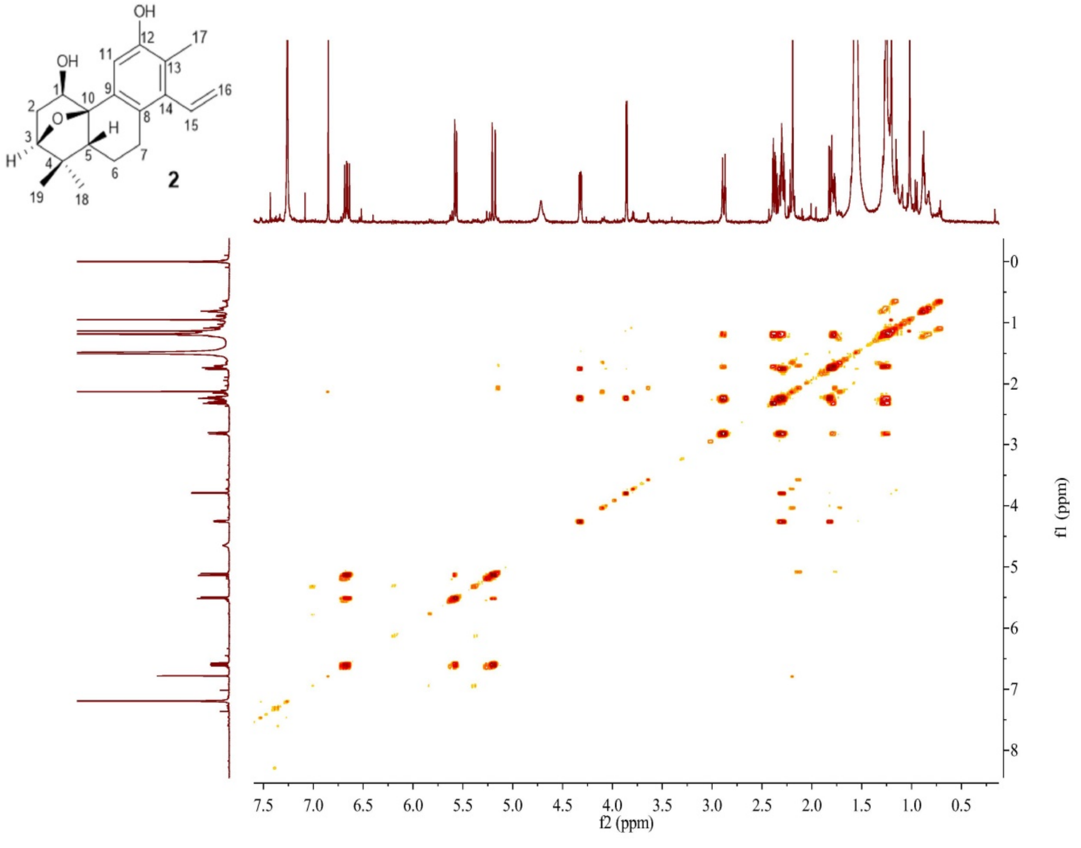

Figure S14 HMBC spectrum of aspidoptoid B (**2**) in $CDCl_3$

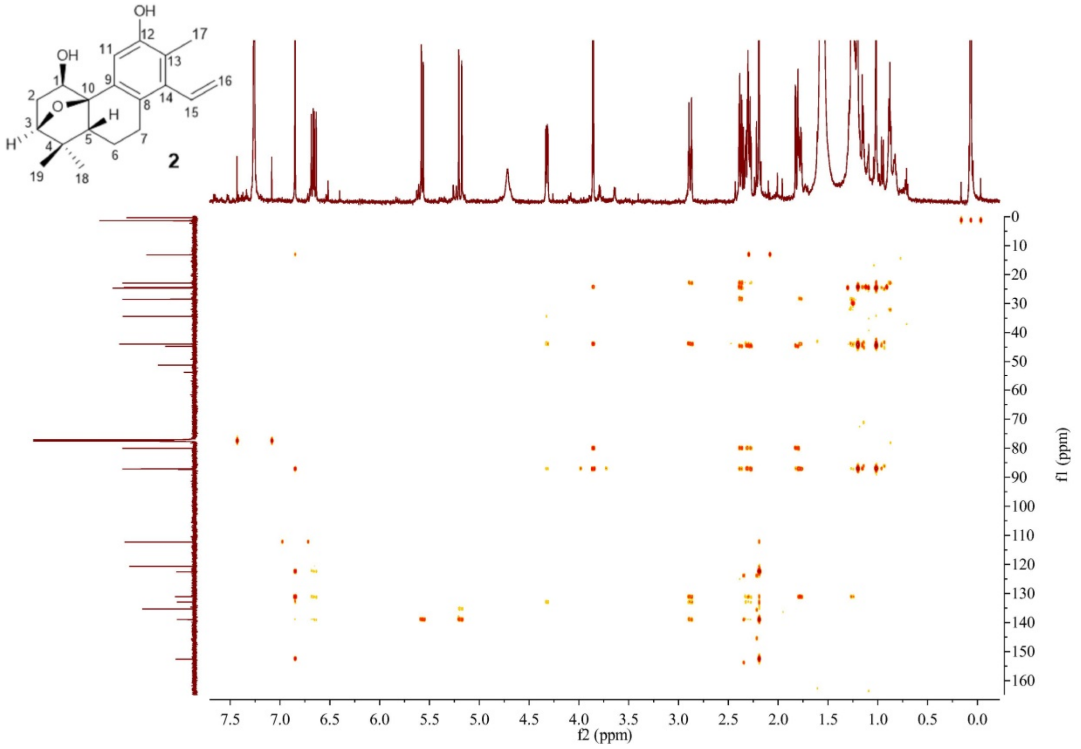

Figure S15 ROESY spectrum of aspidoptoid B (**2**) in $CDCl_3$

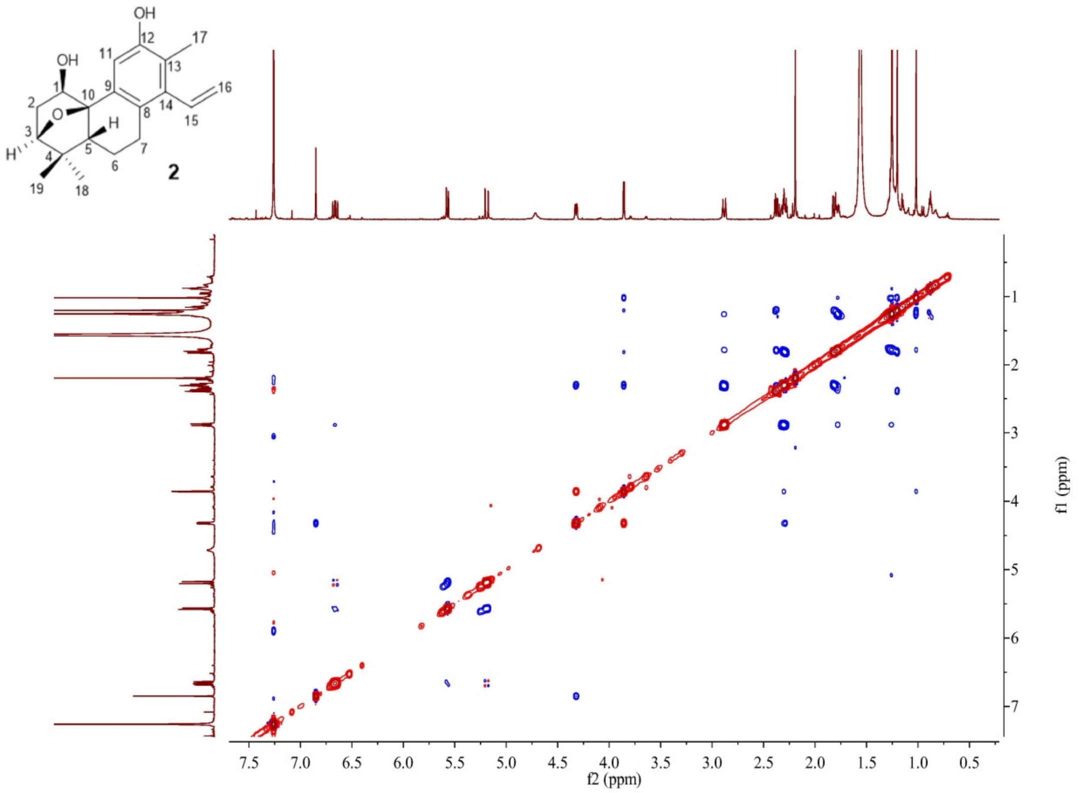

Figure S16 Postitive HR-ESI-MS of aspidoptoid B (**2**)

Formula Predictor Report - sd26.lcd

Page 1 of 1

Data File: E:\DATA\2018\0827\sd26.lcd

Elmt	Val.	Min	Max	Elmt	Val.	Min	Max	Elmt	Val.	Min	Max	Elmt	Val.	Min	Max	Use Adduct
H	1	1	100	O	2	0	10	Si	4	0	0	Br	1	0	0	Na
C	4	10	50	F	1	0	0	S	2	0	0	I	3	0	0	
N	3	0	0	Na	1	0	0	Cl	1	0	0					

Error Margin (ppm):	5	DBE Range:	-2.0 - 100.0	Electron Ions:	both
HC Ratio:	unlimited	Apply N Rule:	yes	Use MSn Info:	yes
Max Isotopes:	all	Isotope RI (%):	1.00	Isotope Res:	10000
MSn Iso RI (%):	75.00	MSn Logic Mode:	AND	Max Results:	10

Event#: 1 MS(E+) Ret. Time : 0.413 Scan# : 63

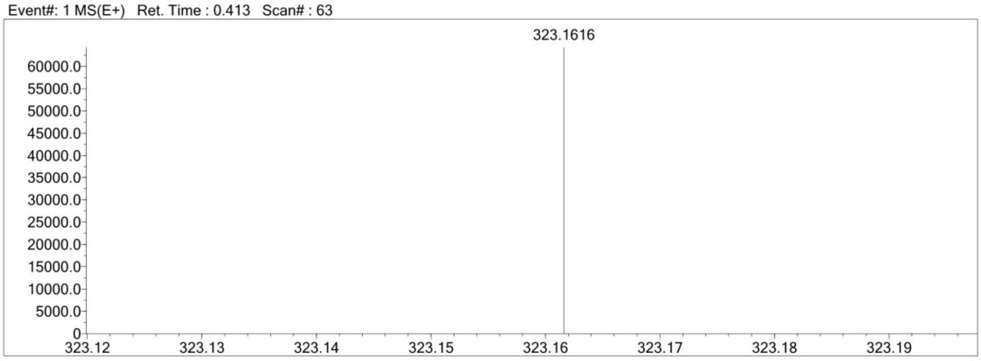

Measured region for 323.1616 m/z

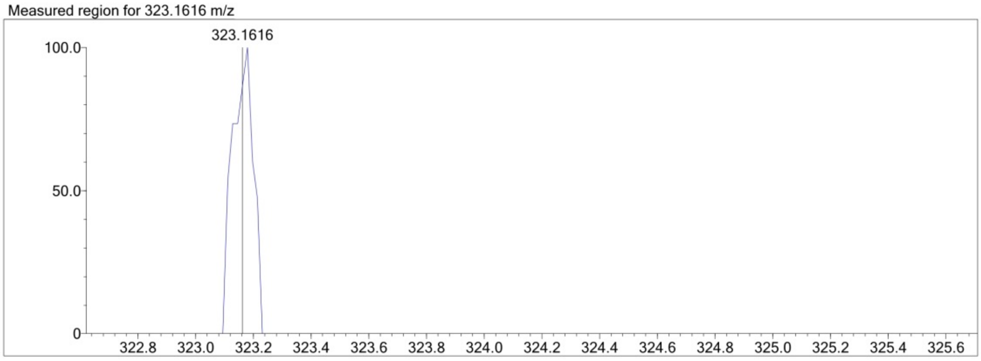

C19 H24 O3 [M+Na]+ : Predicted region for 323.1618 m/z

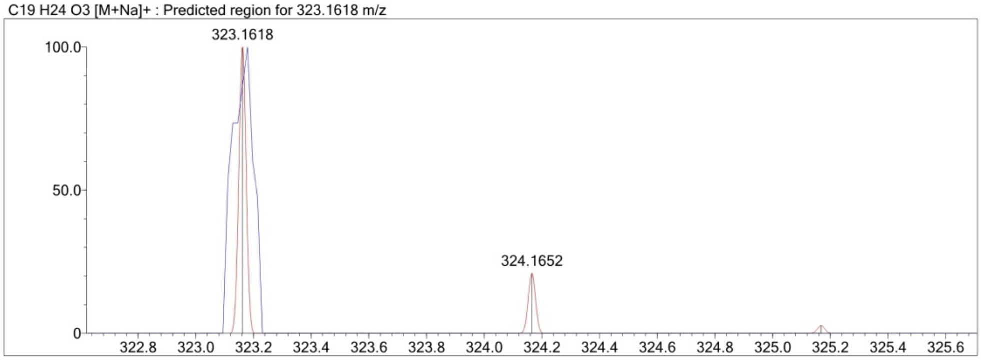

Formula (M)	Ion	Meas. m/z	Pred. m/z	Df. (mDa)	Df. (ppm)	DBE
C19 H24 O3	[M+Na]+	323.1616	323.1618	-0.2	-0.62	8.0

Figure S17 IR spectrum of aspidoptoid B (**2**)

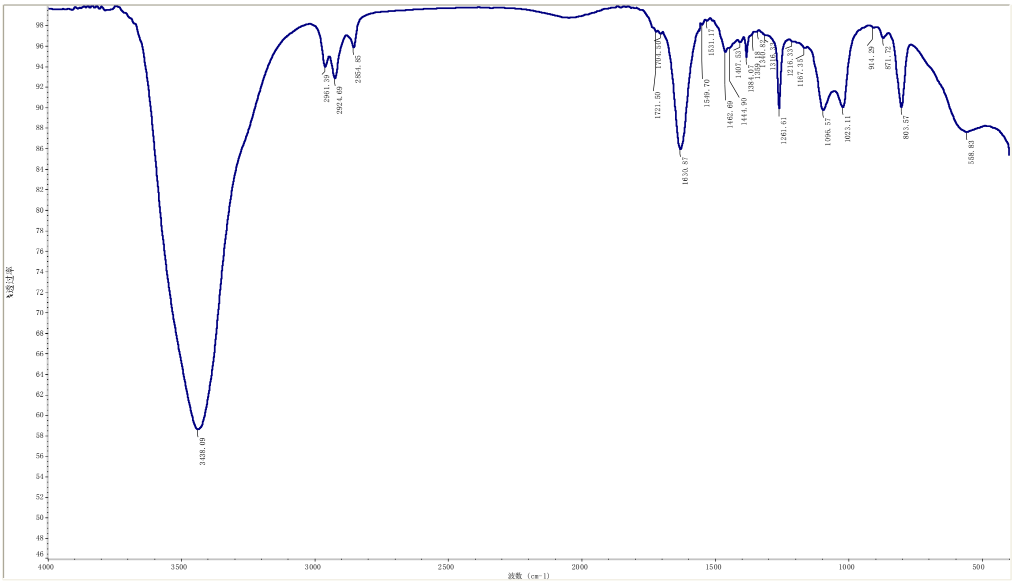

sample name: sd26

KBr压片

采集时间: 星期一 8月 27 15:45:01 2018 (GMT+08:00)

样品扫描次数: 16
背景扫描次数: 16
分辨率: 4.000
采样增益: 1.0
动镜速度: 0.4747
光阑: 80.00

Figure S18 UV spectrum of aspidoptoid B (**2**)

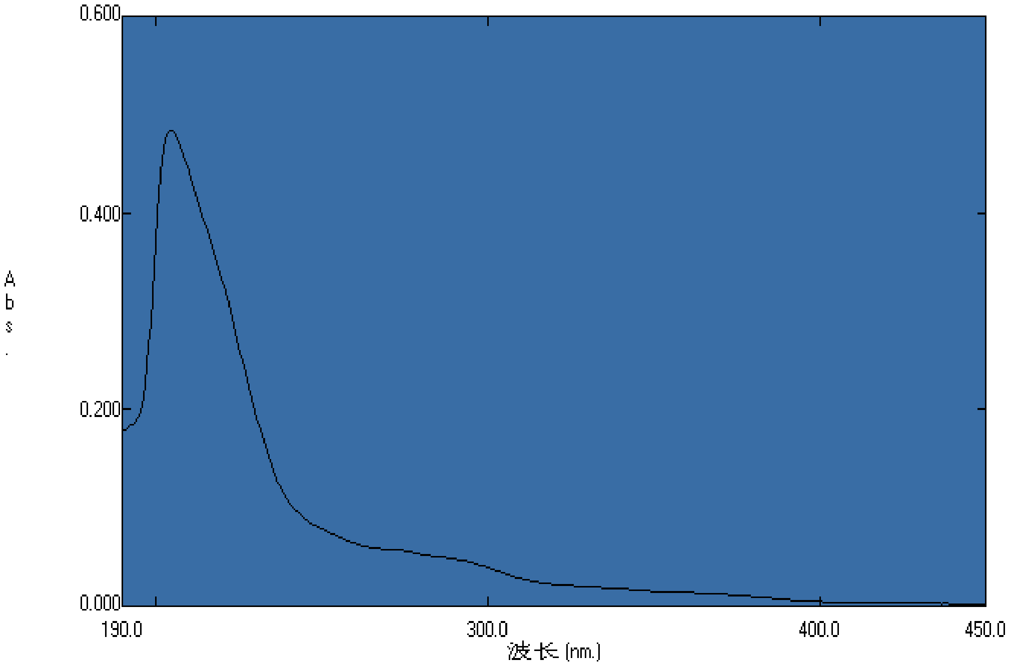

Figure S19 ^{1}H NMR (600 M) spectrum of aspidoptoid C (**3**) in CD_3Cl_3

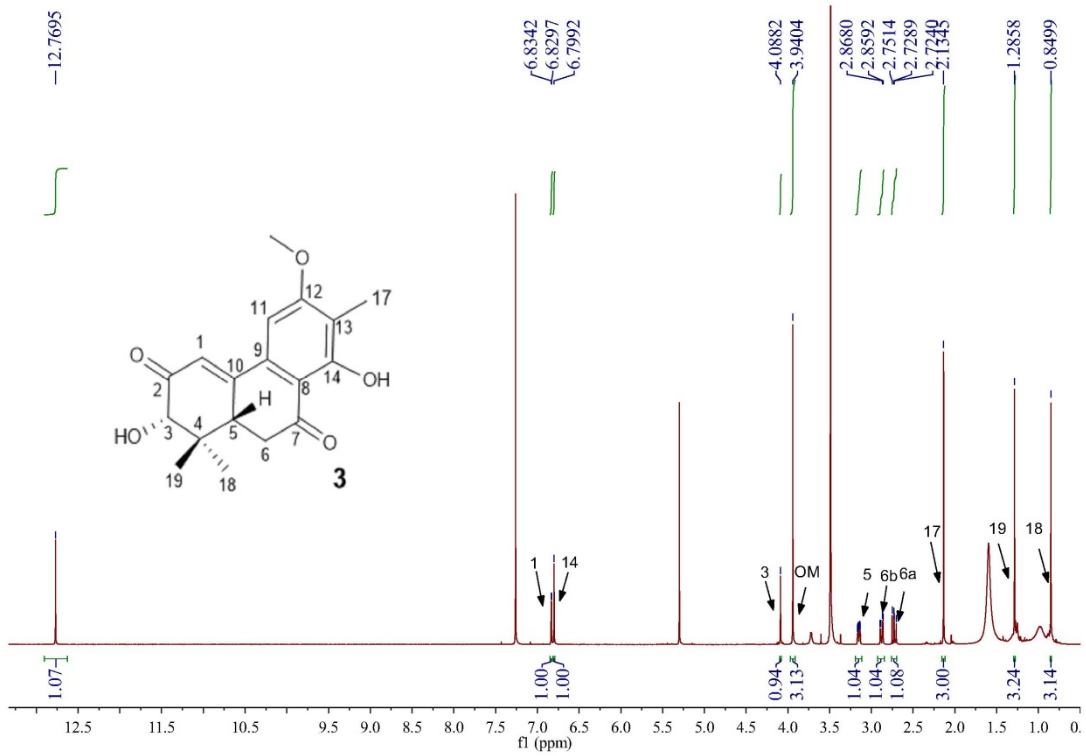

Figure S20 ^{13}C NMR (150 M) spectrum of aspidoptoid C (**3**) in CD_3Cl_3

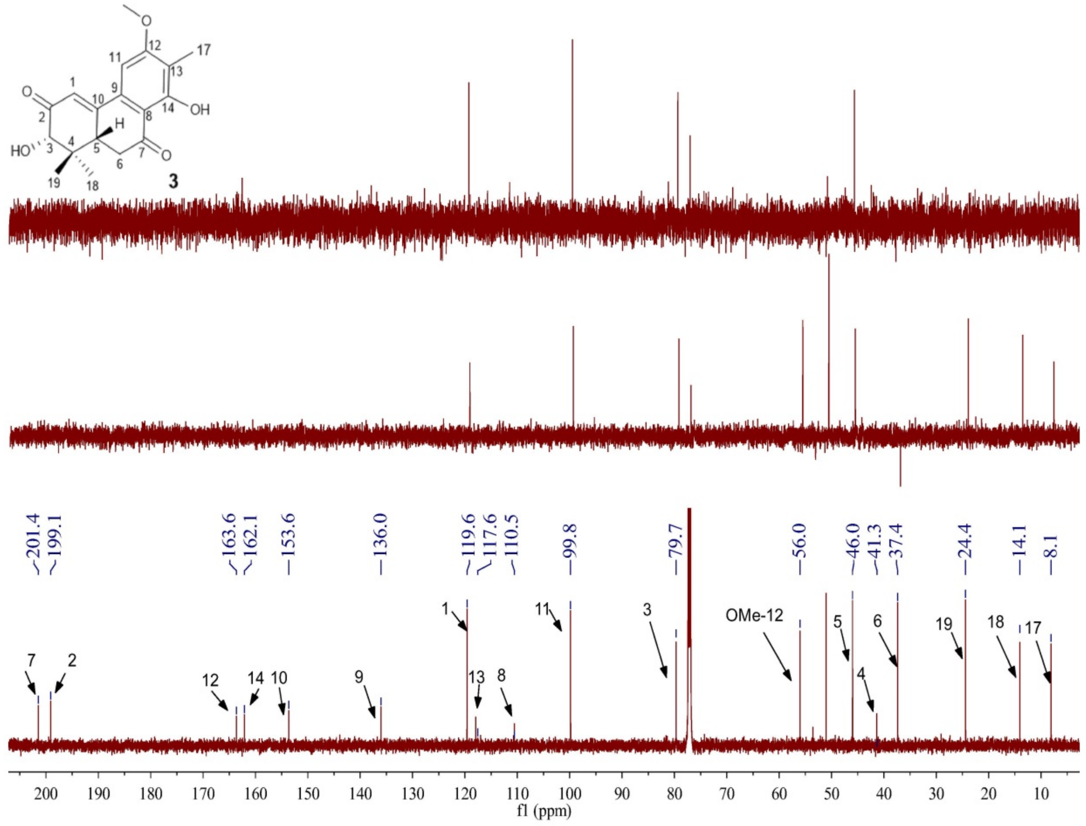

Figure S21 HSQC spectrum of aspidoptoid C (**3**) in CD_3Cl_3

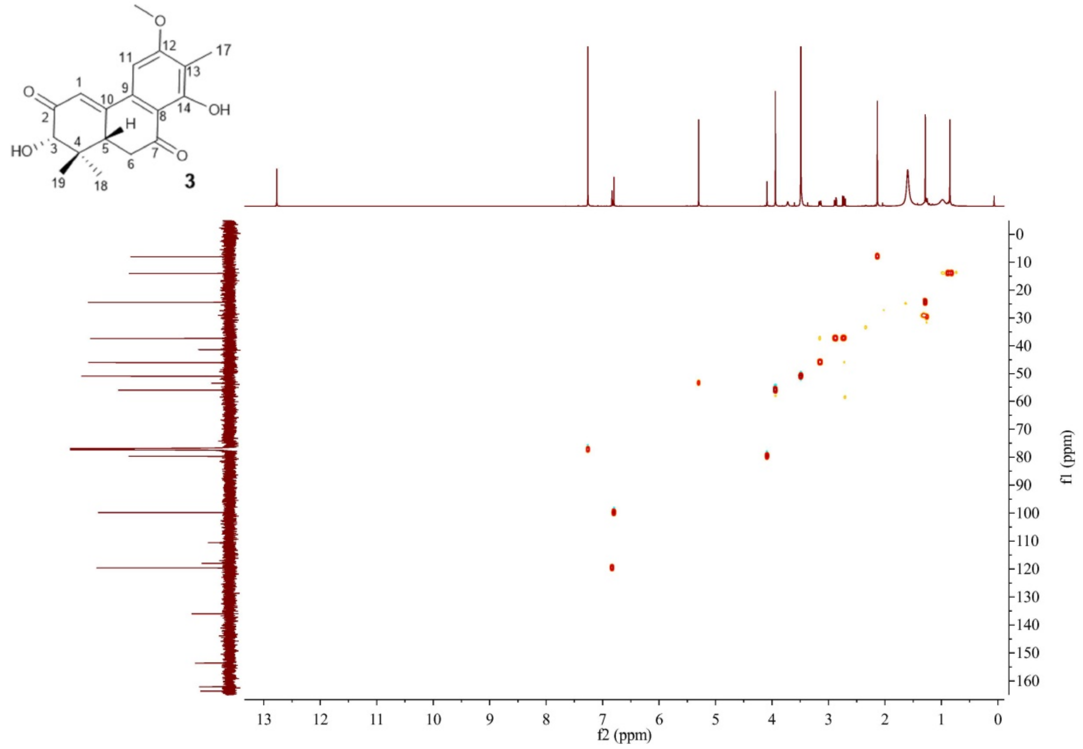

Figure S22 ^1H-^1H COSY spectrum of aspidoptoid C (**3**) in $CDCl_3$

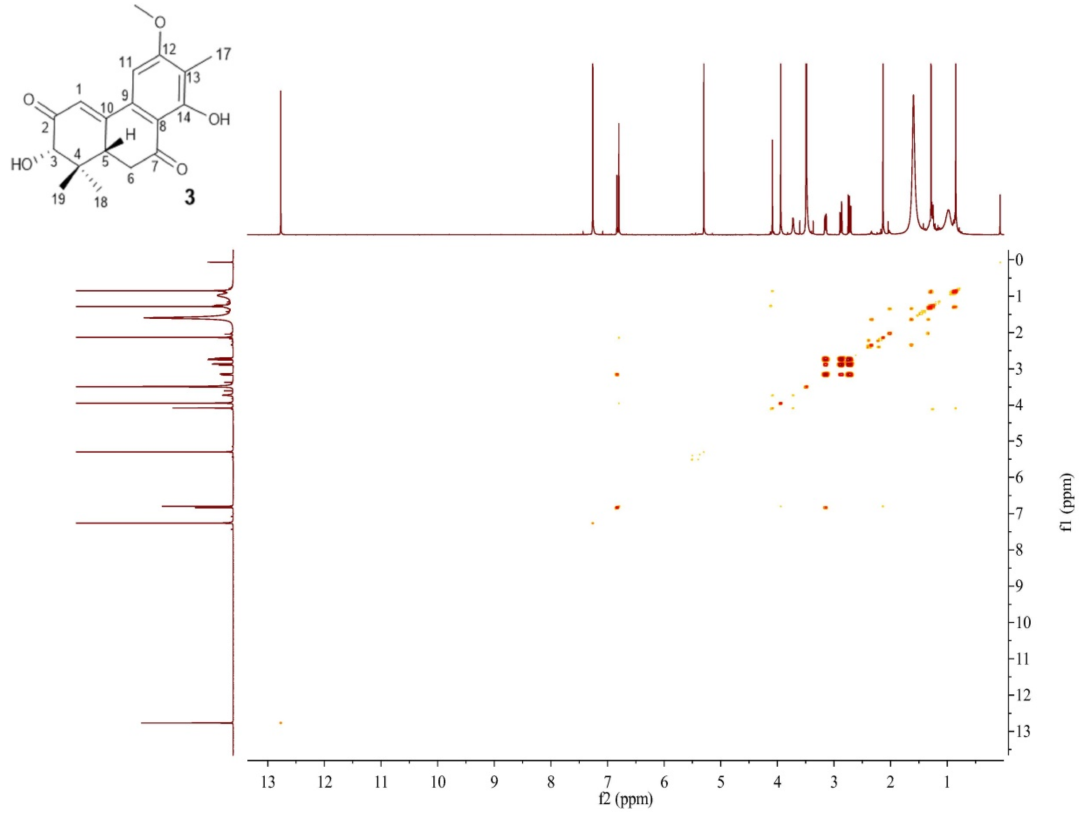

Figure S23 HMBC spectrum of aspidoptoid C (**3**) in CD3Cl3

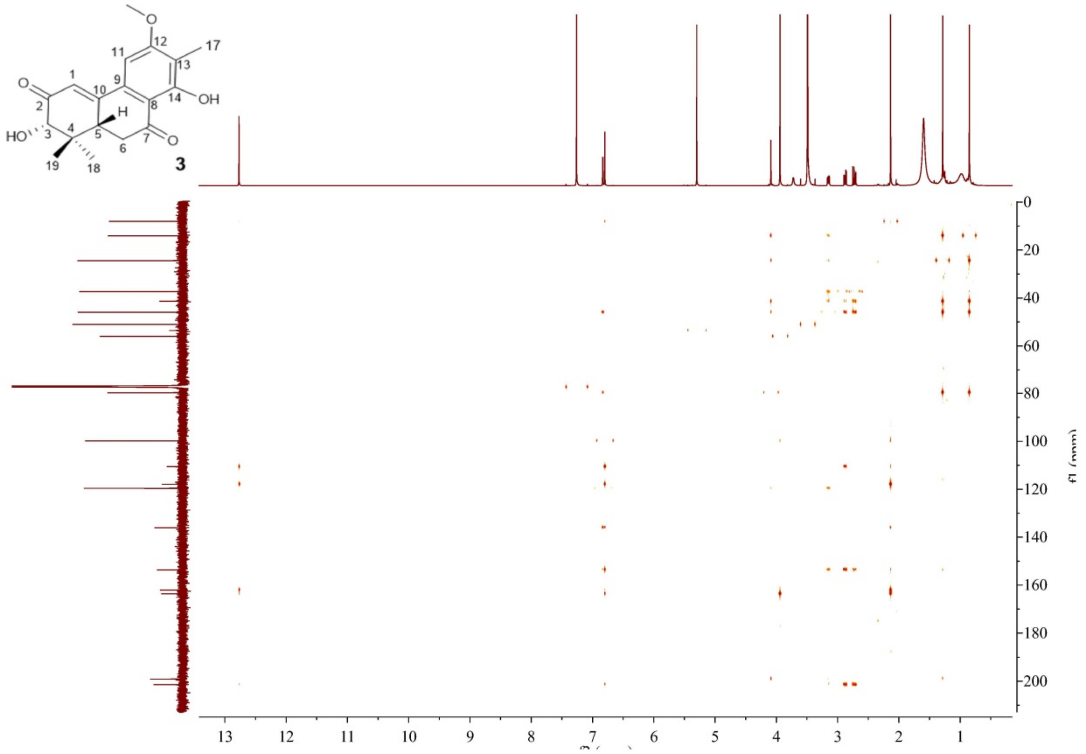

Figure S24 ROESY spectrum of aspidoptoid C (**3**) in CD3Cl3

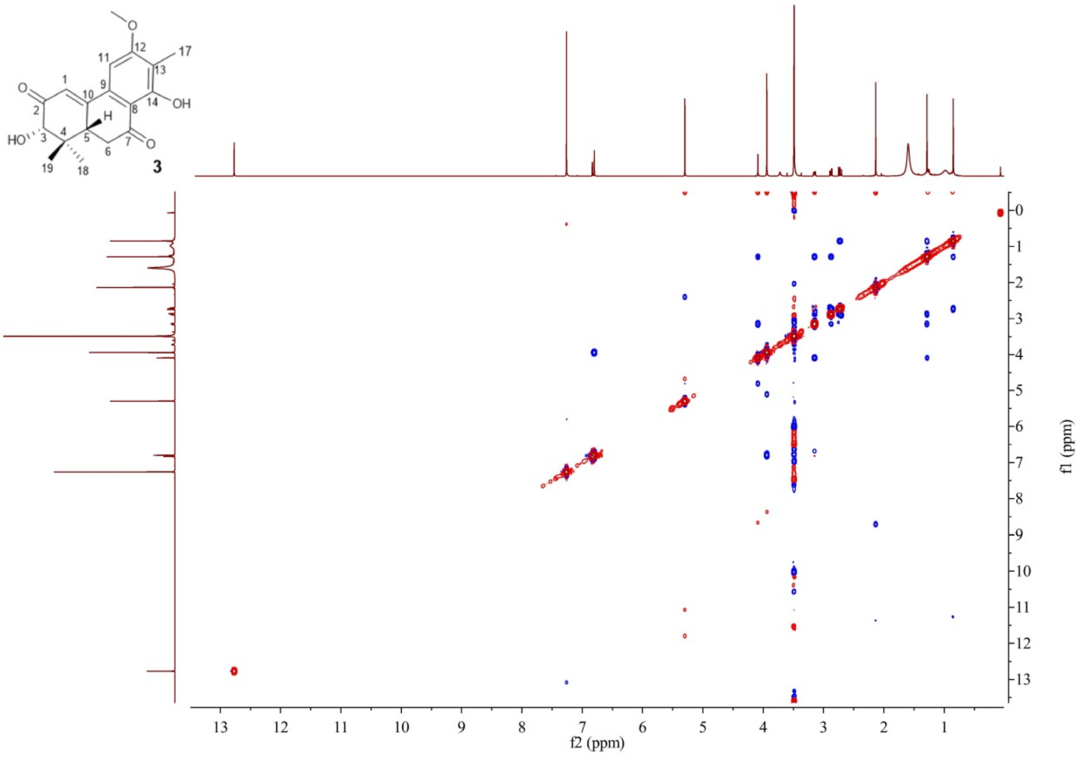

Figure S25 Negative HR-ESI-MS spectrum of aspidoptoid C (**3**)

Formula Predictor Report - sd15.lcd

Page 1 of 1

Data File: E:\DATA\2018\0827\sd15.lcd

Elmt	Val.	Min	Max	Elmt	Val.	Min	Max	Elmt	Val.	Min	Max	Elmt	Val.	Min	Max	Use Adduct
H	1	1	100	O	2	0	10	Si	4	0	0	Br	1	0	0	H
C	4	10	50	F	1	0	0	S	2	0	0	I	3	0	0	
N	3	0	0	Na	1	0	0	Cl	1	0	0					

Error Margin (ppm):	5	DBE Range:	-2.0 - 100.0	Electron Ions:	both
HC Ratio:	unlimited	Apply N Rule:	yes	Use MSn Info:	yes
Max Isotopes:	all	Isotope RI (%):	1.00	Isotope Res:	10000
MSn Iso RI (%):	75.00	MSn Logic Mode:	AND	Max Results:	10

Event#: 2 MS(E-) Ret. Time : 0.373 -> 0.387 Scan# : 58 -> 60

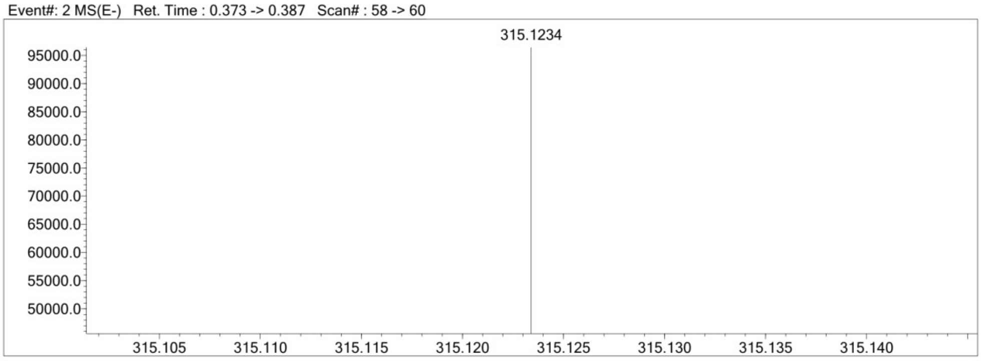

Measured region for 315.1234 m/z

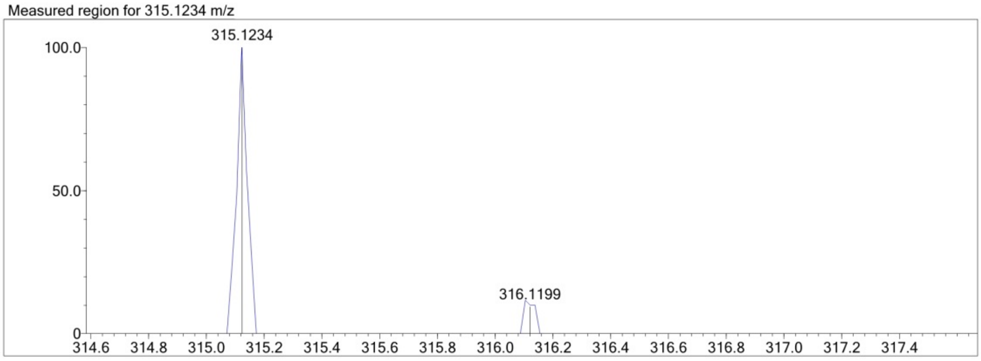

C18 H20 O5 [M-H]- : Predicted region for 315.1238 m/z

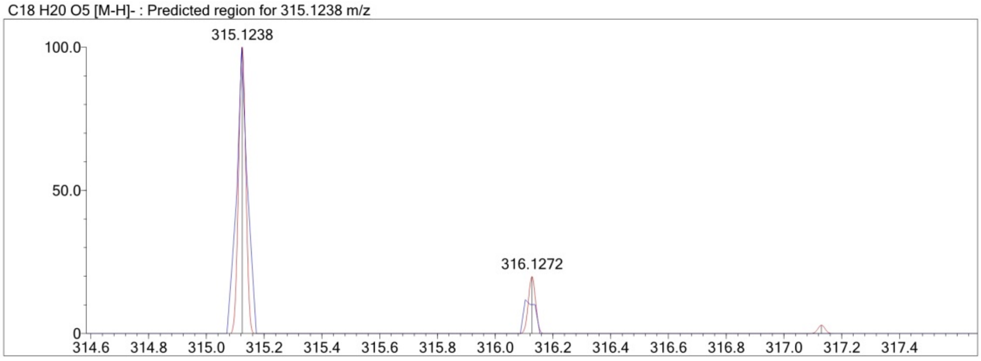

Formula (M)	Ion	Meas. m/z	Pred. m/z	Df. (mDa)	Df. (ppm)	DBE
C18 H20 O5	[M-H]-	315.1234	315.1238	-0.4	-1.27	9.0

Figure S26 IR spectrum of aspidoptoid C (**3**)

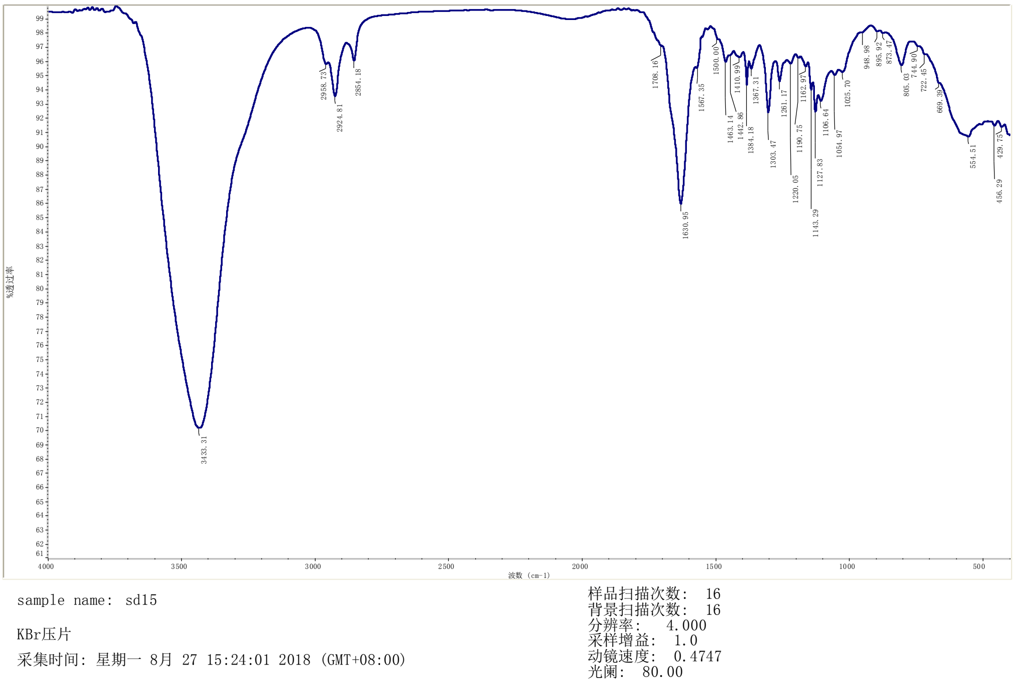

Figure S27 UV spectrum of aspidoptoid C (**3**)

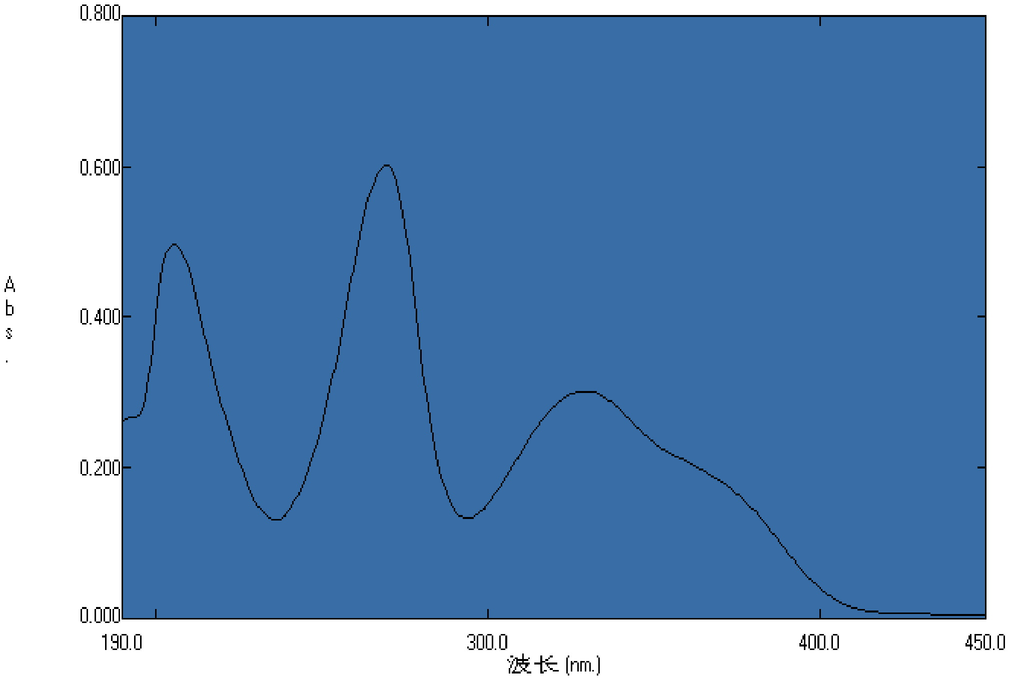

Figure S28 ^{1}H NMR (600 M) spectrum of aspidoptoid D (**4**) in CD_3Cl_3

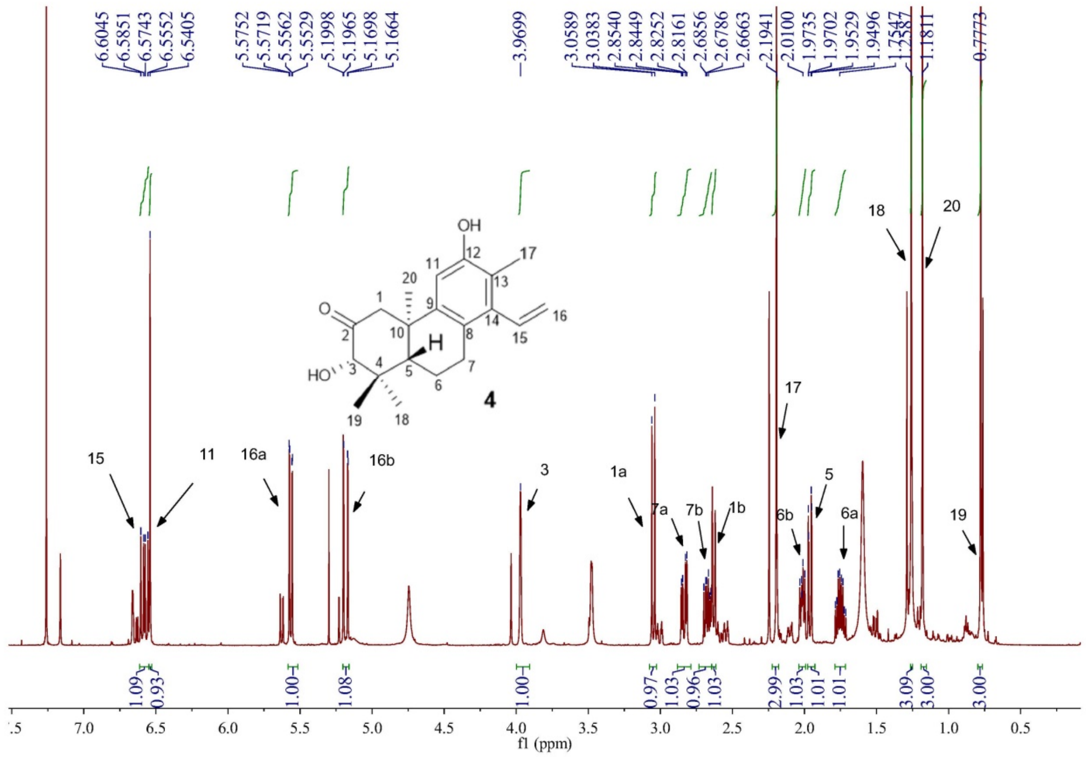

Figure S29 ^{13}C NMR (150 M) spectrum of aspidoptoid D (**4**) in CD_3Cl_3

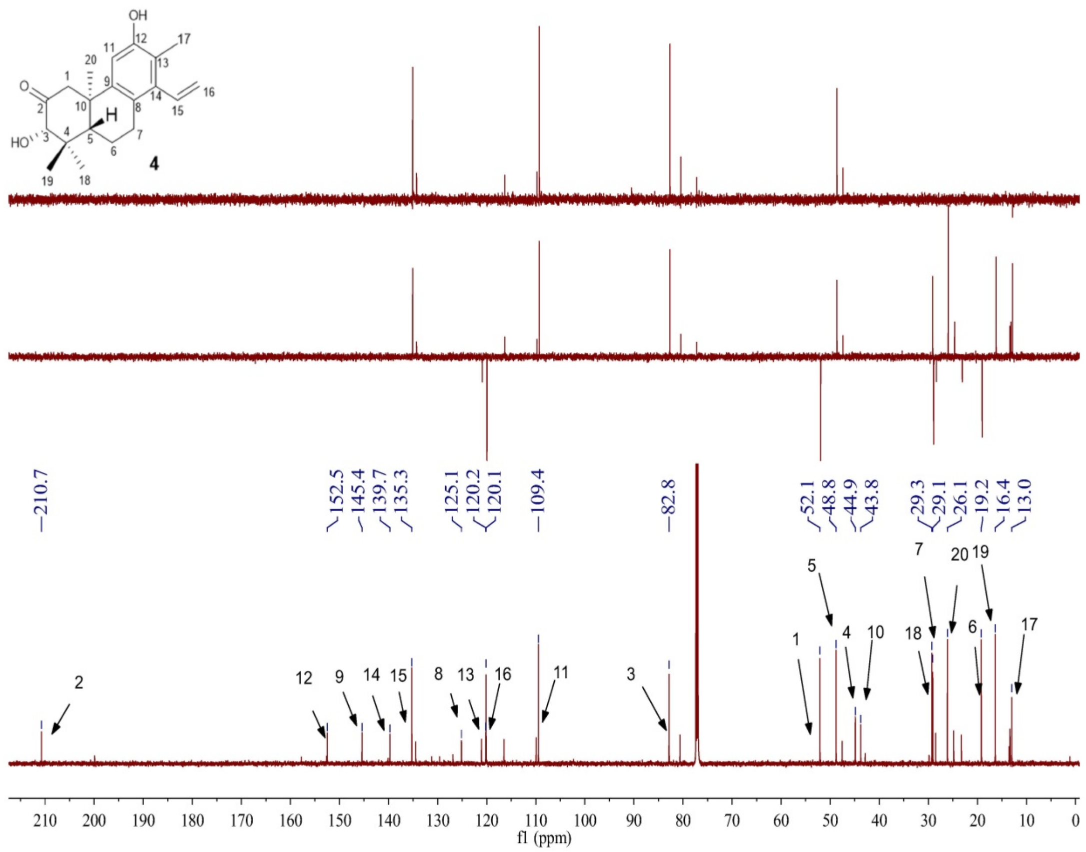

Figure S30 HSQC spectrum of aspidoptoid D (**4**) in CD_3Cl_3

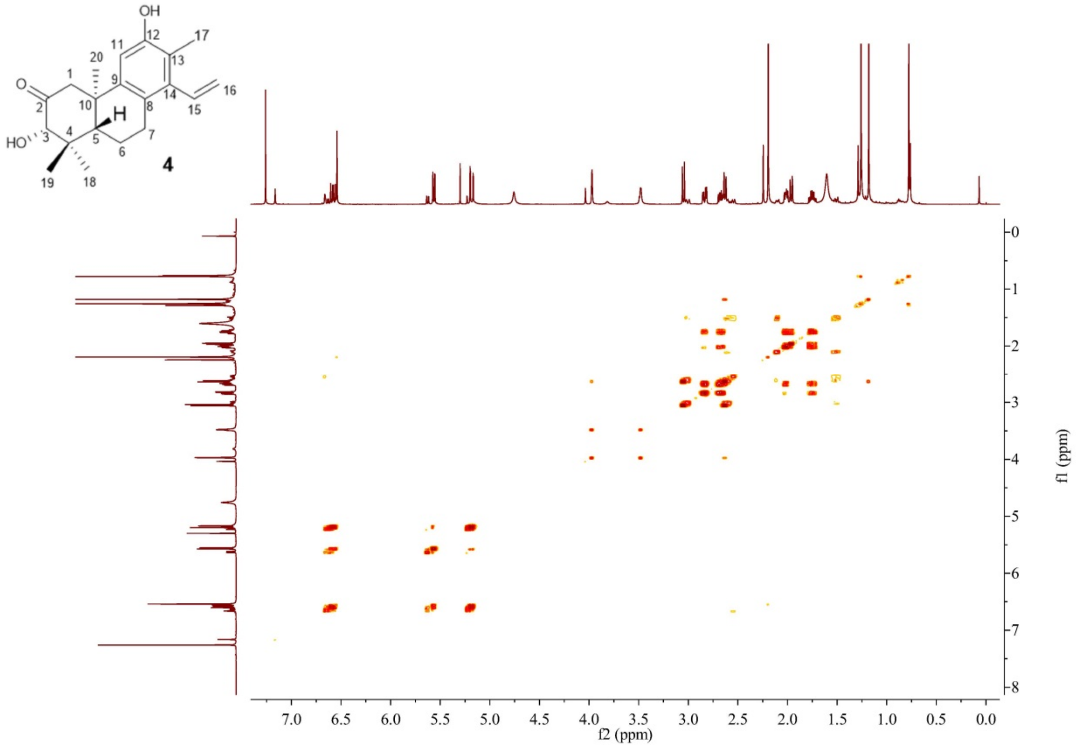

Figure S31 ^{1}H-^{1}H COSY spectrum of aspidoptoid D (**4**) in $CDCl_3$

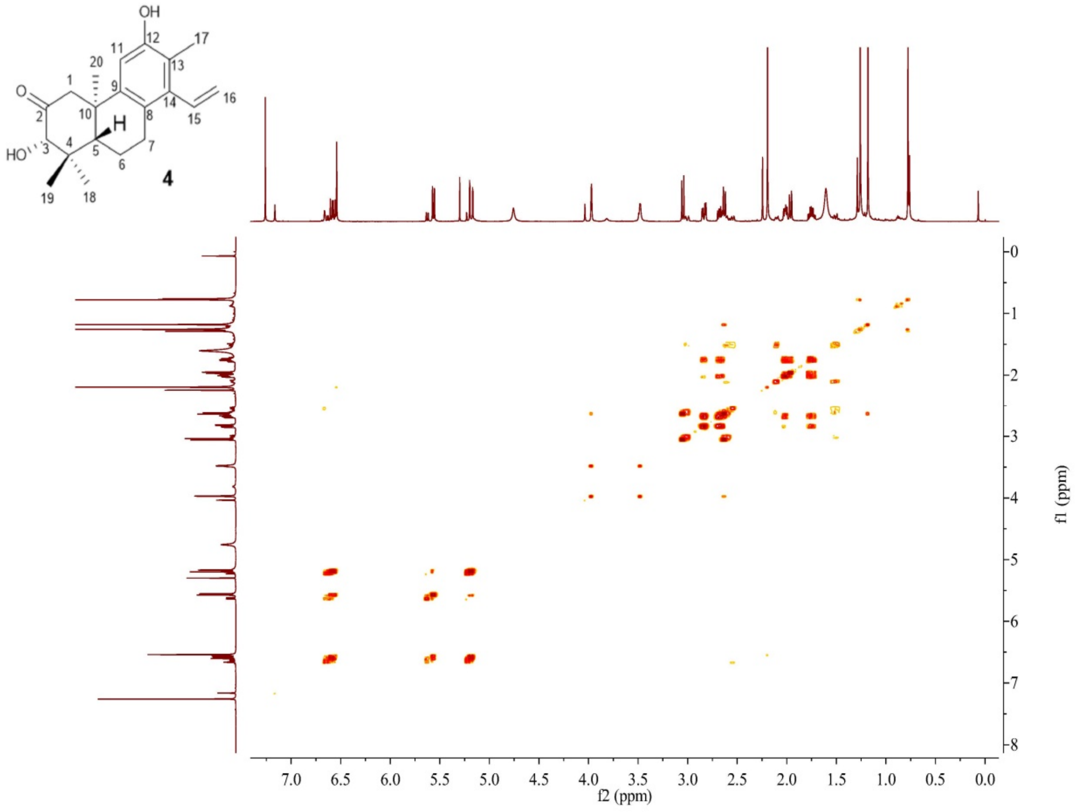

Figure S32 HMBC spectrum of aspidoptoid D (**4**) in CD3Cl3

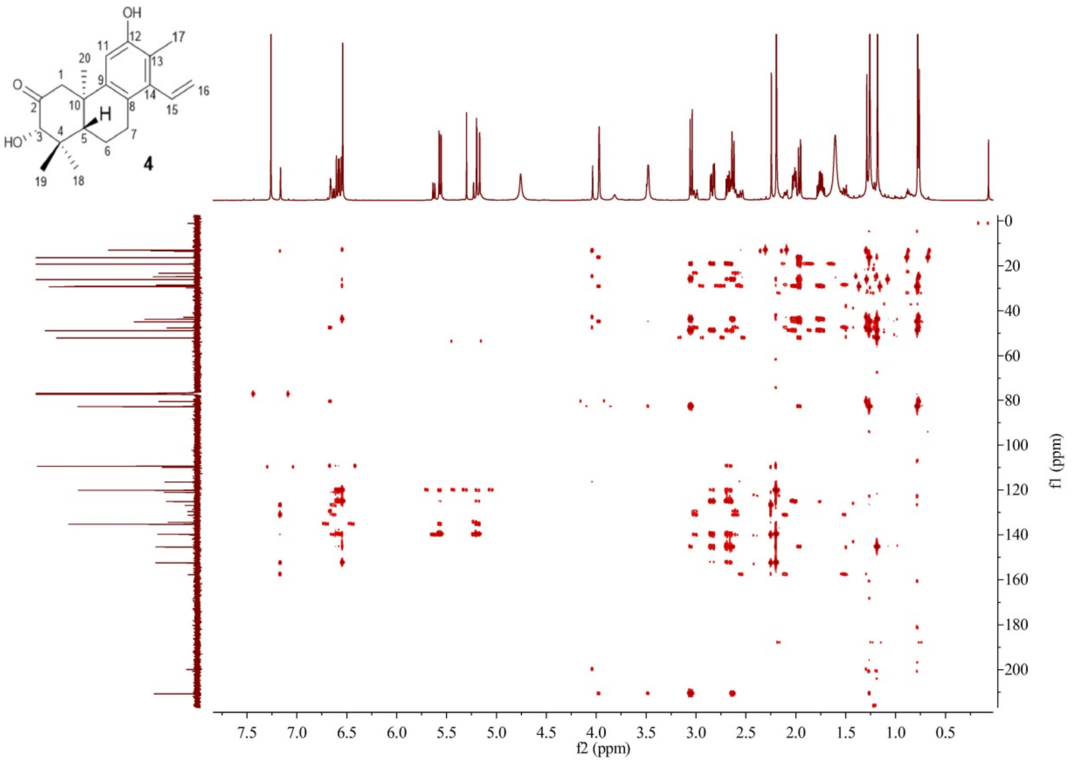

Figure S33 ROESY spectrum of aspidoptoid D (**4**) in CD3Cl3

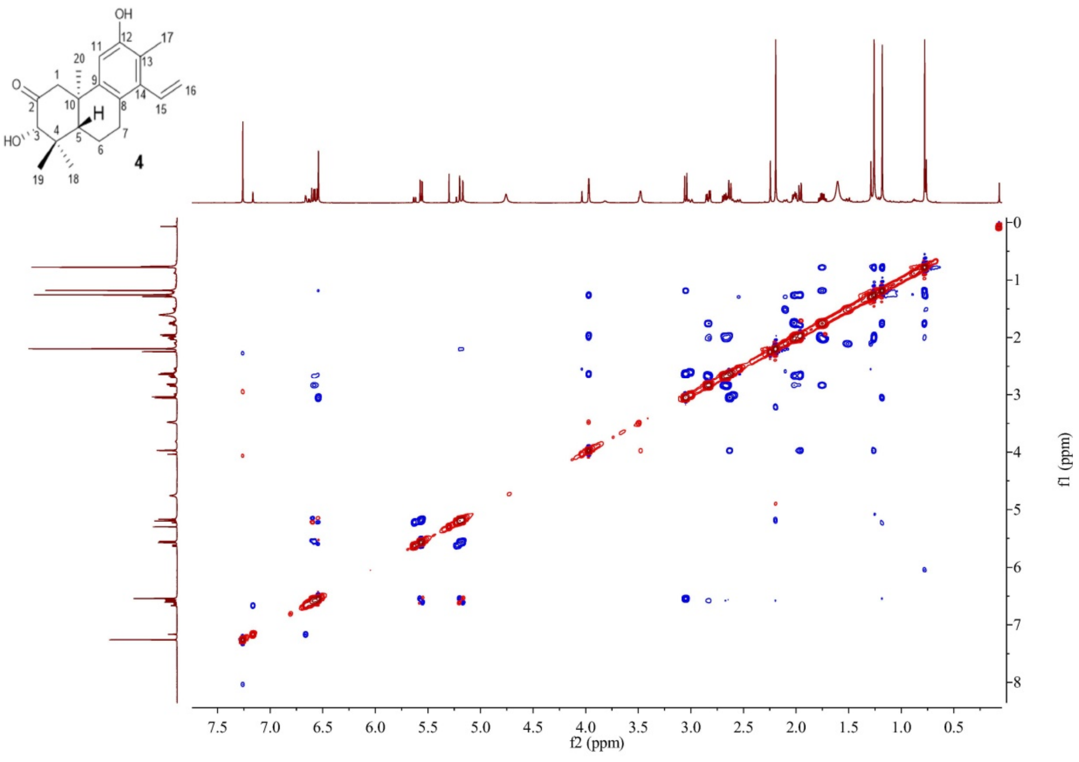

Figure S34 Postive HR-ESI-MS spectrum of aspidoptoid D (**4**)

Formula Predictor Report - sd13.lcd | Page 1 of 1

Data File: E:\DATA\2018\0827\sd13.lcd

Elmt	Val.	Min	Max	Elmt	Val.	Min	Max	Elmt	Val.	Min	Max	Elmt	Val.	Min	Max	Use Adduct
H	1	1	100	O	2	0	10	Si	4	0	0	Br	1	0	0	Na
C	4	10	50	F	1	0	0	S	2	0	0	I	3	0	0	
N	3	0	0	Na	1	0	0	Cl	1	0	0					

Error Margin (ppm):	5	DBE Range:	-2.0 - 100.0	Electron Ions:	both
HC Ratio:	unlimited	Apply N Rule:	yes	Use MSn Info:	yes
Max Isotopes:	all	Isotope RI (%):	1.00	Isotope Res:	10000
MSn Iso RI (%):	75.00	MSn Logic Mode:	AND	Max Results:	10

Event#: 1 MS(E+) Ret. Time : 0.360 -> 0.373 Scan# : 55 -> 57

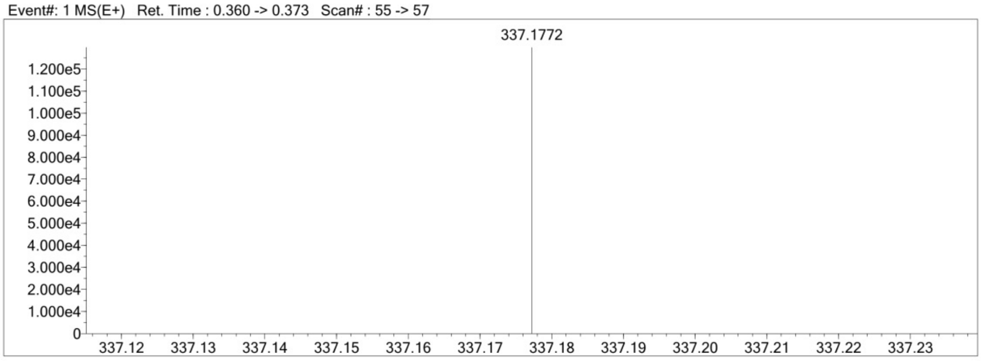

Measured region for 337.1772 m/z

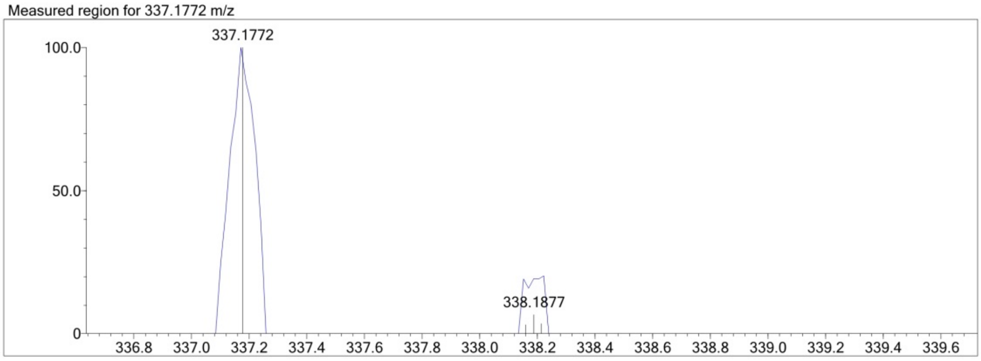

C20 H26 O3 [M+Na]+ : Predicted region for 337.1774 m/z

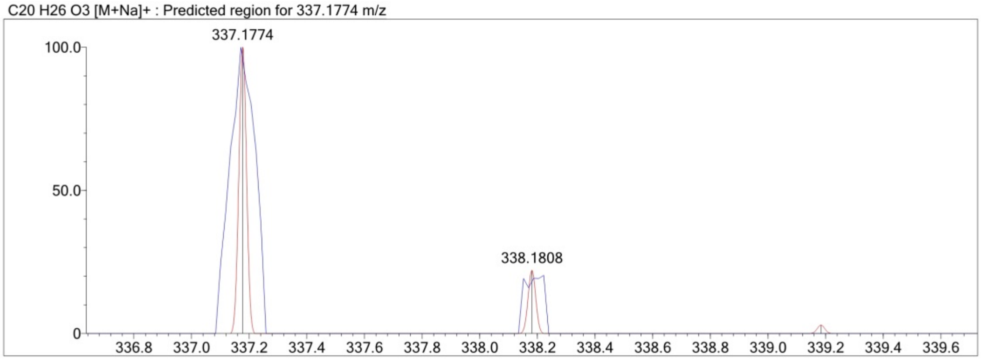

Formula (M)	Ion	Meas. m/z	Pred. m/z	Df. (mDa)	Df. (ppm)	DBE
C20 H26 O3	[M+Na]+	337.1772	337.1774	-0.2	-0.59	8.0

Figure S35 IR spectrum of aspidoptoid D (**4**)

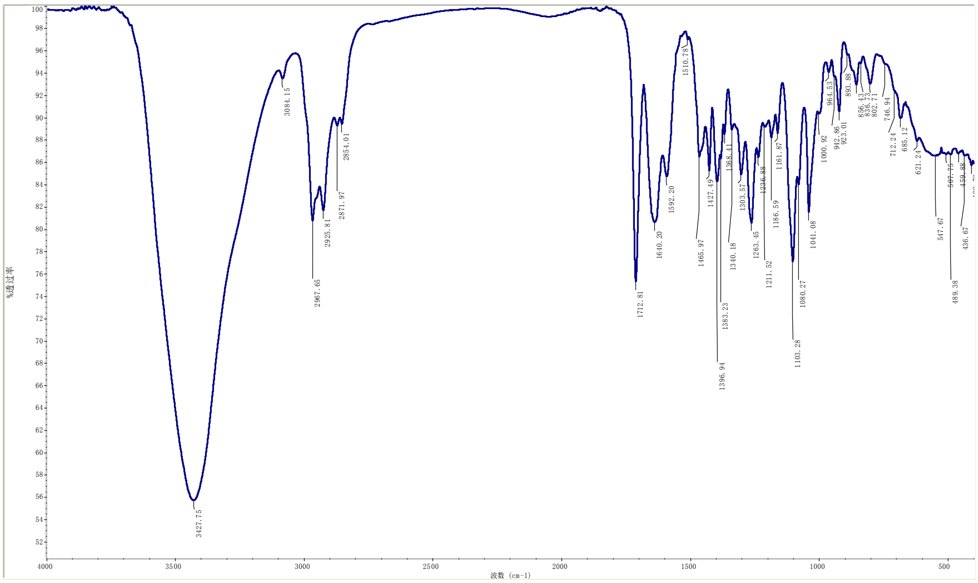

sample name: sd13

KBr压片

采集时间: 星期一 8月 27 11:00:11 2018 (GMT+08:00)

样品扫描次数: 16
背景扫描次数: 16
分辨率: 4.000
采样增益: 1.0
动镜速度: 0.4747
光阑: 80.00

Figure S36 UV spectrum of aspidoptoid D (**4**)

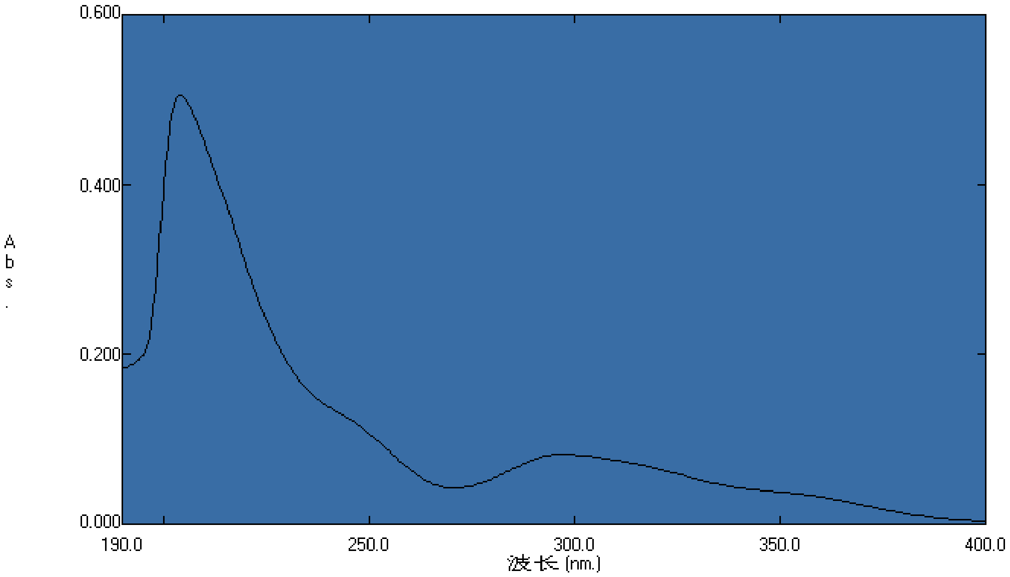

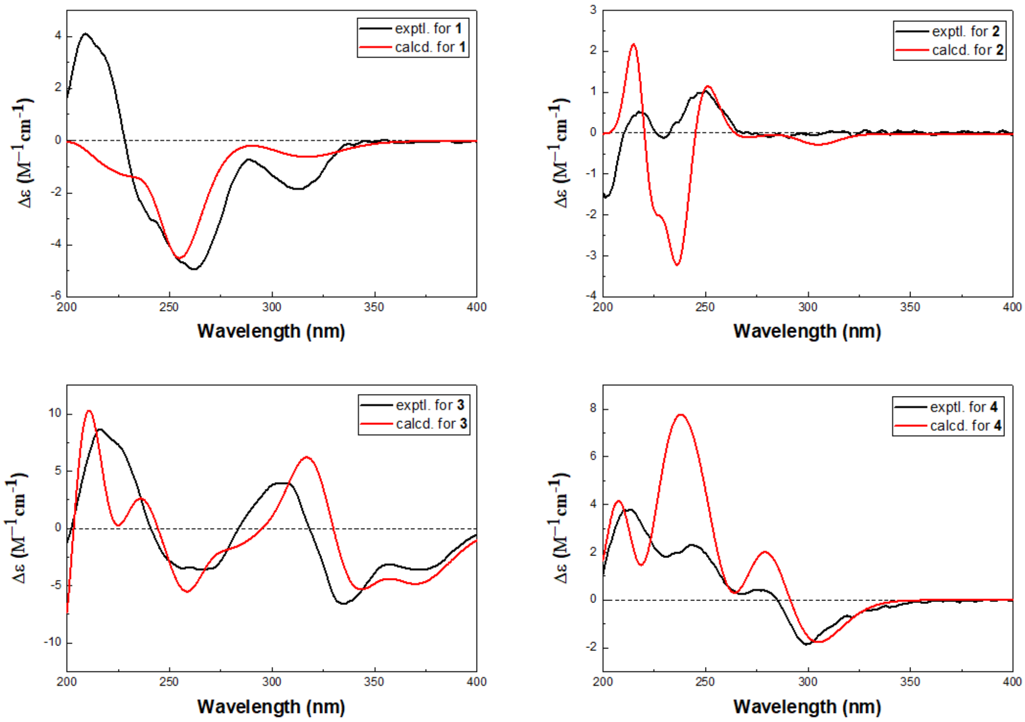

Figure S37 Experimental and B3LYP/6-311+G(2d,2p)//B3LYP/6-31G(d) calculated ECD spectra of **1-4**.

The absolute configuration of compounds **1-4** were determined by quantum chemical TDDFT calculations of their theoretical ECD spectra. Firstly, conformational analysis of compounds **1-4** was carried out via Monte Carlo searching using molecular mechanism with MMFF force field in the Spartan 08 program.[1] In the relative energy window of 0-2 Kcal/mol, the result showed 12 lowest energy conformers for **1**, 6 ones for **2,** 2 ones for **3** and 4 ones for **4**. The conformers were then reoptimized using DFT at the B3LYP/6-31G(d) level in vacuum in the Gaussian 09 program.[2] The B3LYP/6-31G(d) harmonic vibrational frequencies were further calculated to confirm their stability. The energies, oscillator strengths, and rotational strengths of the first about 20 electronic excitations were calculated using the TDDFT methodology at the B3LYP/6-311++G(2d,2p) level in vacuum. The ECD spectra were simulated by the overlapping Gaussian function (σ = 0.20 eV for **1**, **2**, **3** and 0.3 eV for **4**),[3] in which velocity rotatory strengths of the first 22 exited states for **1,** 20 exited states for **2**, 23 exited states for **3** and 20 exited states for **4** were adopted. To get the conformationally averaged ECD spectra, the simulated spectra of the lowest energy conformers were averaged according to the Boltzmann distribution theory and their relative Gibbs free energy (ΔG).

1. *Spartan 08*; Wavefunction Inc.:Irvine, CA.
2. *Gaussian 09*, Revision A.1, Frisch, M. J.; Trucks, G. W.; Schlegel, H. B.; Scuseria, G. E.; Robb, M. A.; Cheeseman, J. R.; Scalmani, G.; Barone, V.; Mennucci, B.; Petersson, G. A.; Nakatsuji, H.; Caricato, M.; Li, X.; Hratchian, H. P.; Izmaylov, A. F.; Bloino, J.; Zheng, G.; Sonnenberg, J. L.; Hada, M.; Ehara, M.; Toyota, K.; Fukuda, R.; Hasegawa, J.; Ishida, M.; Nakajima, T.; Honda, Y.; Kitao, O.; Nakai, H.; Vreven, T.; Montgomery, Jr., J. A.; Peralta, J. E.; Ogliaro, F.; Bearpark, M.; Heyd, J. J.; Brothers, E.; Kudin, K. N.; Staroverov, V. N.; Kobayashi, R.; Normand, J.; Raghavachari, K.; Rendell, A.; Burant, J. C.; Iyengar, S. S.; Tomasi, J.; Cossi, M.; Rega, N.; Millam, J. M.; Klene, M.; Knox, J. E.; Cross, J. B.; Bakken, V.; Adamo, C.; Jaramillo, J.; Gomperts, R.; Stratmann, R. E.; Yazyev, O.; Austin, A. J.; Cammi, R.; Pomelli, C.; Ochterski, J. W.; Martin, R. L.; Morokuma, K.; Zakrzewski, V. G.; Voth, G. A.; Salvador, P.; Dannenberg, J. J.; Dapprich, S.; Daniels, A. D.; Farkas, Ö.; Foresman, J. B.; Ortiz, J. V.; Cioslowski, J.; Fox, D. J. Gaussian, Inc., Wallingford CT, 2009.
3. Stephens, P. J.; Harada, N. ECD cotton effect approximated by the Gaussian curve and other methods. *Chirality* 2010, *22*, 229–233.

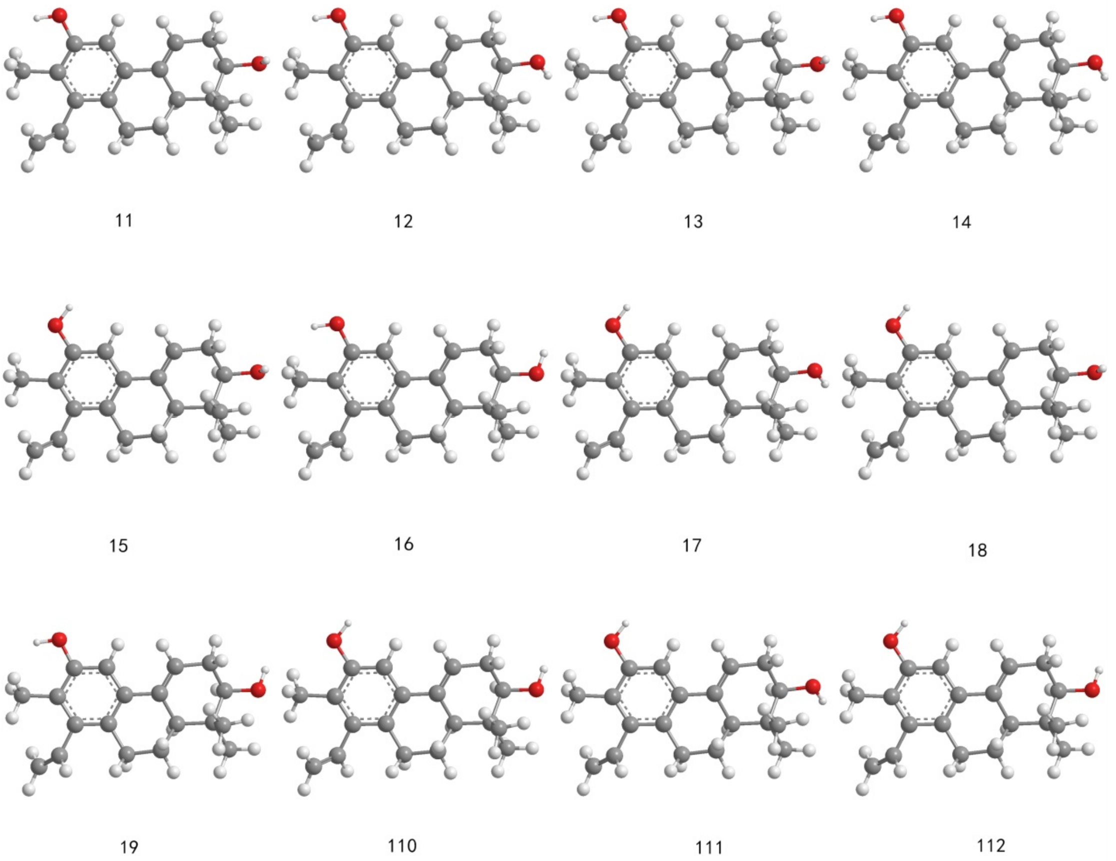

Figure S38 B3LYP/6-311+G(d) optimized lowest energy 3D conformers of **1**.

Energy analysis for 1

conf.	Gibbs free energy (298.15 K)		
	G (Hartree)	ΔG (Kcal/mol)	Boltzmann Distribution
11	-888.84026625	1.32403877	0.03902424
12	-888.84114974	0.76964040	0.09953575
13	-888.83837497	2.51083494	0.00525816
14	-888.83927215	1.94784596	0.01360753
15	-888.84135754	0.63924393	0.12405759
16	-888.84063033	1.09557511	0.05739973
17	-888.84237624	0.00000000	0.36517513
18	-888.83958240	1.75316114	0.01890507
19	-888.83888164	2.19289470	0.00899578
110	-888.84173459	0.40264147	0.18499860
111	-888.84053703	1.15412175	0.05199560
112	-888.84005047	1.45944277	0.03104683

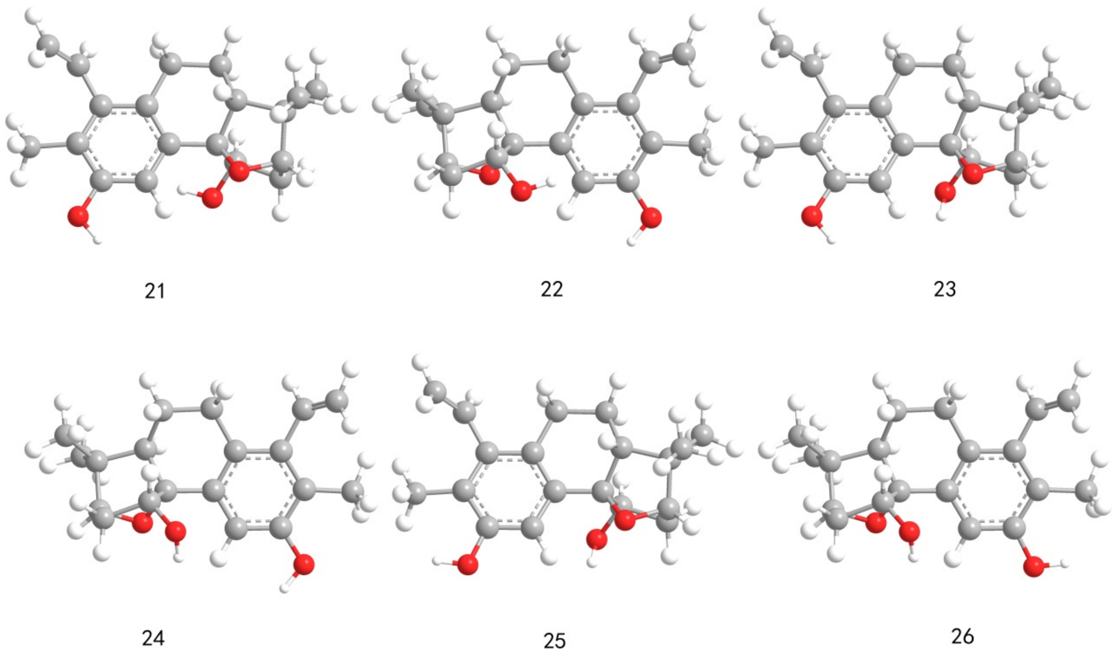

Figure S39 B3LYP/6-311+G(d) optimized lowest energy 3D conformers of **2**.

Energy analysis for 2

conf.	Gibbs free energy (298.15 K)		
	G (Hartree)	ΔG (Kcal/mol)	Boltzmann Distribution
21	-964.0455061	0.165091474	0.249980929
22	-964.0457692	0	0.330369175
23	-964.045371	0.249849182	0.216639728
24	-964.0439055	1.16945808	0.045836966
25	-964.0449206	0.532479461	0.134410173
26	-964.0432451	1.583896729	0.022763028

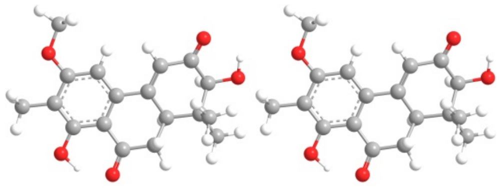

31

32

Figure S40 B3LYP/6-311+G(d) optimized lowest energy 3D conformers of **3**.

Energy analysis for 3

conf.	Gibbs free energy (298.15 K)		
	G (Hartree)	ΔG (Kcal/mol)	Boltzmann Distribution
31	-1074.074255	0	0.500609392
32	-1074.074252	0.001443272	0.499390608

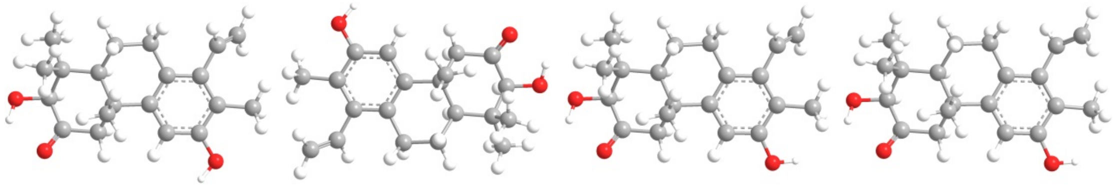

41

42

43

44

Figure S41 B3LYP/6-311+G(d) optimized lowest energy 3D conformers of **4**.

Energy analysis for 4

conf.	Gibbs free energy (298.15 K)		
	G (Hartree)	ΔG (Kcal/mol)	Boltzmann Distribution
41	-1003.39	0.161722	0.389498
42	-1003.39	0	0.511831
43	-1003.38	1.502415	0.040469
44	-1003.38	1.28726	0.058202

ECD Data for 1-4:

State	11		12		13		14		15		16	
	Excitation energies(eV)	Rotatory Strengths*	Excitation energies(eV)	Rotatory Strengths*	Excitation energies(eV)	Rotatory Strengths*	Excitation energies(eV)	Rotatory Strengths*	Excitation energies(eV)	Rotatory Strengths*	Excitation energies(eV)	Rotatory Strengths*
1	6.0844	1.6653	6.0725	-0.2179	6.0755	-8.4368	6.0547	-6.1167	6.0725	-2.1312	6.0904	-2.8869
2	6.0606	-4.1107	6.0107	-0.5555	6.0429	7.0522	6.0370	-8.3250	6.0253	6.1538	6.0019	-2.0016
3	5.9558	6.0239	5.9530	-26.3848	6.0136	-7.1302	5.9817	1.9686	6.0019	-14.3940	5.9501	6.0843
4	5.9330	-23.9282	5.9444	-6.4454	5.9874	1.3707	5.9615	4.1450	5.9501	-5.1138	5.9387	-29.4365
5	5.8879	6.9367	5.9104	11.4357	5.9104	-3.4775	5.8684	19.1157	5.8963	-18.9376	5.9104	-0.5263
6	5.8380	-6.7408	5.8243	1.2966	5.8216	-1.5910	5.8189	-4.6411	5.8546	-4.7866	5.8824	5.7462
7	5.8025	1.3631	5.8107	0.9192	5.7863	2.6657	5.7648	-2.2271	5.7648	-3.4235	5.7755	-4.8041
8	5.7594	-2.1522	5.6933	2.9438	5.6855	6.6702	5.7381	6.7552	5.7222	-2.8131	5.7728	-4.2948
9	5.6312	-25.6497	5.6210	4.0644	5.6287	13.2393	5.6985	-1.8949	5.6415	-15.5374	5.6751	2.2956
10	5.5755	2.5330	5.5982	-15.0344	5.5831	4.6199	5.5831	-2.6779	5.6236	-21.0776	5.6007	-9.6524
11	5.5531	-4.3356	5.5755	-1.5677	5.5580	-7.3779	5.5630	-12.9812	5.5655	1.1601	5.5730	10.4613
12	5.4965	29.1361	5.4892	-14.5027	5.5111	-1.0148	5.4819	4.3373	5.4819	34.6246	5.5160	-13.6565
13	5.4553	-0.5271	5.4674	35.3648	5.4626	-37.1897	5.4458	-23.2955	5.4386	1.9461	5.4650	13.4971
14	5.4196	9.1781	5.3843	2.0343	5.4148	7.7094	5.3610	5.5140	5.3726	25.9545	5.4243	11.5611
15	5.3196	3.5257	5.2766	3.6195	5.2901	-6.9364	5.2609	0.5261	5.2788	0.7452	5.2453	1.7147
16	5.2233	-1.1253	5.1992	-5.5006	5.2101	8.4253	5.1818	10.1123	5.1240	-5.2132	5.2057	-3.8725
17	5.1261	-6.1880	5.0263	-5.0355	5.1008	4.5584	5.0243	2.6967	5.0716	-6.4186	5.0799	-4.9142
18	4.9109	13.0512	4.8915	25.8679	4.9859	-100.6463	4.9779	-101.7750	4.9462	101.2837	4.8973	31.5789
19	4.8742	87.1667	4.8704	72.0242	4.8800	-31.7088	4.8551	-36.0444	4.8025	21.5595	4.8761	66.3817
20	4.6845	-10.5450	4.6863	-12.3143	4.7546	-2.4692	4.7510	-0.1973	4.7005	-16.7514	4.6863	-10.4309
21	4.4765	-11.8969	4.4749	-10.2959	4.5206	15.2664	4.5190	12.3385	4.4830	-17.0876	4.4830	-12.9101
22	4.0273	-4.2825	4.0234	-5.1449	4.0709	-10.1111	4.0669	-9.9955	4.0129	-8.2485	4.0312	-4.9884

State	17		18		19		110		111		112	
	Excitation energies(eV)	Rotatory Strengths*	Excitation energies(eV)	Rotatory Strengths*	Excitation energies(eV)	Rotatory Strengths*	Excitation energies(eV)	Rotatory Strengths*	Excitation energies(eV)	Rotatory Strengths*	Excitation energies(eV)	Rotatory Strengths*
1	6.0547	-1.6522	6.0904	7.8093	6.0576	2.4436	6.0904	1.9325	6.0636	-0.2174	6.0370	-13.5523
2	6.0136	-28.6324	6.0399	-4.8851	6.0078	-6.0624	6.0282	-5.2970	5.9903	2.6432	6.0165	18.7835
3	5.9817	0.6659	6.0019	-9.2521	5.9817	3.5589	5.9788	-2.2340	5.9701	2.2989	6.0048	3.2960
4	5.9444	5.8177	5.9615	5.5608	5.9472	-1.5529	5.9558	-19.5947	5.9472	-3.2734	5.9273	2.3695
5	5.9189	-0.8912	5.9132	-7.0127	5.8991	6.4862	5.8935	2.8419	5.8907	13.8460	5.8991	7.6300
6	5.8963	-9.5543	5.8796	7.4070	5.8824	2.9993	5.8824	-21.0031	5.8768	-2.0448	5.8796	-6.4366
7	5.7275	-0.9004	5.7328	5.7335	5.7890	7.9033	5.7836	-2.5070	5.7541	-0.7193	5.7944	7.8748
8	5.7011	1.9583	5.6595	-11.9173	5.7701	-3.5041	5.7407	-1.2692	5.7090	-0.1809	5.7621	-6.3342
9	5.6185	3.9336	5.6338	2.6131	5.6492	-1.7583	5.6855	4.1110	5.6777	0.3611	5.6338	-1.6232
10	5.5856	-30.7730	5.6058	5.9463	5.6007	9.7394	5.5881	-17.5146	5.5831	-0.9621	5.5856	-2.7962
11	5.5580	-11.8080	5.5680	-1.2646	5.5705	-1.9417	5.5431	-8.6907	5.5332	5.9954	5.5456	7.9427
12	5.5258	-4.7301	5.4867	1.2723	5.5283	-13.4814	5.5209	-7.9261	5.5087	-24.5070	5.5357	-17.9958
13	5.4267	50.2653	5.4291	22.8840	5.4553	-19.2328	5.4795	15.7599	5.3983	-2.8543	5.4172	32.9360
14	5.3703	12.3368	5.3866	-41.7468	5.4125	1.6965	5.3819	33.6492	5.3471	-2.7637	5.3843	-44.1526
15	5.2365	0.7633	5.2321	-2.6490	5.2189	-4.1678	5.1861	2.0507	5.2035	1.7793	5.1581	-2.2367
16	5.1050	-11.3250	5.1367	-7.4190	5.2013	4.4325	5.1134	-2.7240	5.1261	-6.8443	5.1261	-19.7162
17	4.9839	-1.3681	5.0530	-5.3023	5.0633	0.6871	5.0366	-7.9447	5.0427	-108.5442	5.0448	-108.4653
18	4.9422	96.0669	5.0489	-108.4106	4.9839	-96.3158	4.9422	101.2315	4.9779	-6.8627	5.0223	3.9408
19	4.7988	24.0955	4.8174	-34.6709	4.8647	-31.5376	4.7840	19.9546	4.8137	-36.7050	4.8081	-30.3291
20	4.6970	-18.2645	4.7437	10.0962	4.7583	-2.1941	4.6916	-16.8970	4.7328	10.1378	4.7256	7.4103

21	4.4879	-16.7886	4.5206	16.0037	4.5222	12.4315	4.4895	-18.8930	4.5239	13.1182	4.5222	13.0403
22	4.0091	-9.1981	4.0523	-9.8734	4.0696	-10.7634	4.0155	-9.1668	4.0470	-9.9712	4.0496	-10.7274

State	**21**		**22**		**23**		**24**		**25**		**26**	
	Excitation energies(eV)	Rotatory Strengths*	Excitation energies(eV)	Rotatory Strengths*	Excitation energies(eV)	Rotatory Strengths*	Excitation energies(eV)	Rotatory Strengths*	Excitation energies(eV)	Rotatory Strengths*	Excitation energies(eV)	Rotatory Strengths*
1	6.1661	-10.1601	6.2062	8.9782	6.1661	-28.8610	6.1692	1.6674	6.1845	2.7081	6.1938	-9.5458
2	6.1356	33.0550	6.1508	10.4293	6.1477	15.6279	6.1661	6.9315	6.1753	0.5323	6.1265	-1.8280
3	6.1204	13.1009	6.0994	14.8015	6.1114	3.8928	6.1054	-26.3851	6.1477	20.2922	6.1144	-3.7418
4	6.0844	-12.0730	6.0576	22.2877	6.0576	33.3951	6.1045	6.4621	6.0934	4.2456	6.1054	-2.1171
5	6.0517	19.9144	6.0282	-30.5662	6.0429	-8.1038	6.0282	-15.3113	6.0636	-8.6238	6.0844	13.8341
6	5.9759	-6.5915	5.9615	5.5104	6.0341	-2.4862	5.9846	3.2548	6.0606	-14.0386	6.0517	-29.8225
7	5.9444	-4.0435	5.9415	-7.0523	5.8824	-16.5231	5.8851	12.7827	5.9615	-14.5284	5.9530	1.7746
8	5.9132	-1.9072	5.9245	-42.7720	5.8518	4.0020	5.8298	0.3990	5.9020	-1.7938	5.8879	-1.8939
9	5.9104	-7.8436	5.8991	8.1876	5.7755	-32.4239	5.7971	6.8294	5.8025	-17.3448	5.8518	1.4744
10	5.7674	8.2768	5.8161	10.4170	5.7169	10.3010	5.7301	-12.3541	5.7461	-2.1984	5.7487	-7.2350
11	5.7487	3.1081	5.7090	36.4872	5.6134	-41.1361	5.6466	12.5707	5.6647	0.0862	5.6751	-1.9095
12	5.6338	-10.6219	5.6389	-1.9725	5.5881	0.0301	5.5605	-20.0561	5.6007	-0.3913	5.5831	11.1773
13	5.5906	-48.8911	5.5780	-9.2215	5.5209	-17.5760	5.5283	9.5232	5.5655	-36.5342	5.5357	-1.2714
14	5.4989	-13.2657	5.4940	1.6981	5.5160	0.6642	5.4746	15.1897	5.4940	3.3941	5.4819	-7.7766
15	5.4314	-4.3888	5.4172	15.8921	5.4577	-4.4974	5.4243	7.2012	5.4314	-3.6462	5.4482	13.9283
16	5.3703	9.5364	5.4101	-2.3482	5.3379	3.8509	5.3680	8.5907	5.3310	6.6541	5.3356	4.2546
17	5.1992	-8.4203	5.2211	32.6292	5.1071	1.6206	5.1538	17.7442	5.1431	-21.9281	5.1796	48.6055
18	4.9960	3.4010	5.0121	2.5419	4.9226	-14.4750	4.9226	42.4126	4.9541	13.4552	4.9699	-19.1728

19	4.9501	-31.8288	4.9109	39.9101	4.8800	-22.0875	4.8589	3.8263	4.8992	-31.6884	4.8857	37.3958
20	4.3372	-10.0592	4.3341	10.7036	4.2877	-12.4069	4.3221	9.7339	4.2818	-15.8964	4.3221	16.1090

State	31		32		41		42		43		44	
	Excitation energies(eV)	Rotatory Strengths*	Excitation energies(eV)	Rotatory Strengths*	Excitation energies(eV)	Rotatory Strengths*	Excitation energies(eV)	Rotatory Strengths*	Excitation energies(eV)	Rotatory Strengths*	Excitation energies(eV)	Rotatory Strengths*
1	7.0158	-0.0286	7.0193	-0.0240	6.9951	-7.4762	6.9574	-2.2121	7.0402	-56.8301	7.0332	-34.9843
2	6.8401	-24.1448	6.8401	-24.1252	6.9540	-10.8051	6.9302	33.9911	7.0089	10.6209	6.9608	-0.9805
3	6.8040	-8.8998	6.8040	-8.9166	6.8999	8.6605	6.8665	-38.2216	6.9336	0.5067	6.9033	9.3996
4	6.7587	2.8752	6.7587	2.8718	6.8434	9.1404	6.8106	15.0437	6.8966	-4.9924	6.8599	15.1325
5	6.6761	5.2293	6.6761	5.1068	6.8040	8.6775	6.7587	7.9344	6.7619	-33.4524	6.8204	30.5465
6	6.6542	-9.7219	6.6542	-9.6019	6.6918	-21.2369	6.7362	23.2817	6.7235	14.3929	6.7299	-20.8598
7	6.5924	26.0065	6.5924	26.0377	6.6386	2.0102	6.6448	8.3520	6.6542	-1.9403	6.6231	-0.2469
8	6.5528	-10.8449	6.5528	-10.9033	6.5347	-19.8812	6.5741	-8.3718	6.5710	7.4285	6.5619	1.3421
9	6.4516	6.4464	6.4516	6.4016	6.5108	-10.3721	6.4899	-0.2135	6.5018	-5.0452	6.5227	-0.0075
10	6.4137	14.6541	6.4137	14.7266	6.4224	0.1928	6.4137	13.2080	6.4692	-10.2529	6.4487	4.4188
11	6.2776	12.9825	6.2776	13.0012	6.2749	-9.8217	6.2888	-4.7702	6.3506	-14.0957	6.3139	9.9625
12	6.2638	4.1345	6.2638	4.1311	6.1847	13.0261	6.2063	-0.1663	6.2036	9.6460	6.2555	-14.6882
13	6.2117	-14.7214	6.2117	-14.7323	6.1739	-2.3901	6.1793	17.3560	6.1578	11.8658	6.2009	16.1386
14	5.7513	9.8032	5.7513	9.8240	5.9514	-0.2223	5.9589	20.1951	5.9714	-22.4462	5.9764	48.5209
15	5.4826	-9.9343	5.4826	-9.9606	5.7235	-26.4484	5.6641	46.3591	5.7374	7.7372	5.7304	-4.4422
16	5.3972	21.5630	5.3992	21.5500	5.6126	2.1083	5.5862	21.3009	5.7120	6.2991	5.6709	15.3281
17	5.3223	-32.2071	5.3223	-32.1828	5.4911	-32.3463	5.4911	-19.6590	5.6215	-51.2660	5.5818	12.7097
18	4.8547	-9.6513	4.8547	-9.6510	5.0642	-38.1523	5.0390	-29.0706	5.0914	-21.5808	5.0624	-15.1655

19	4.8054	3.9433	4.8054	3.9515	5.0283	72.1170	5.0018	68.9374	5.0301	66.2612	5.0141	55.3404
20	4.2300	67.6430	4.2300	67.6443	4.8797	-40.6891	4.8814	-30.1147	4.9391	-58.8325	4.9357	-27.0348
21	4.1029	-85.9881	4.1029	-86.0405								
22	3.9346	38.7966	3.9346	38.8437								
23	3.7796	-38.0070	3.7796	-38.0194								

* R(velocity) 10**-40 erg-esu-cm
